# A review of parameter settings for galvanic vestibular stimulation in clinical applications

**DOI:** 10.3389/fnhum.2025.1518727

**Published:** 2025-02-03

**Authors:** Yishai Valter, Linda Vataksi, Aaron R. Allred, Jeffrey R. Hebert, Tad T. Brunyé, Torin K. Clark, Jorge Serrador, Abhishek Datta

**Affiliations:** ^1^Research and Development Soterix Medical Inc., Woodbridge, NJ, United States; ^2^Department of Biomedical Engineering, City College of New York, New York City, NY, United States; ^3^U. S. Army DEVCOM Soldier Center, Natick, MA, United States; ^4^Marcus Institute for the Brain Health, Department of Physical Medicine and Rehabilitation School of Medicine, University of Colorado, Aurora, CO, United States; ^5^Department of Aerospace Engineering Sciences, University of Colorado, Boulder, CO, United States; ^6^Center for Applied Brain and Cognitive Sciences, Tufts University, Medford, MA, United States; ^7^MARCS Institute for Brain, Behaviour and Development, Western Sydney University, Sydney, NSW, Australia

**Keywords:** GVS, galvanic vestibular stimulation, vestibular system, balance disorder, parameters optimization, post-stroke, spatial orientation, motion sickness

## Abstract

Galvanic Vestibular Stimulation (GVS) is a method of manipulating the vestibular system through non-invasive electrical current. Depending on how GVS is applied, it produces specific sensations related to vestibular mediated central pathways. The method has been tested for decades for both medical and non-medical applications and has demonstrated promise in treating a variety of disorders including peripheral vestibular conditions, central vestibular pathology due to neurodegenerative diseases, and post-stroke motor rehabilitation. As GVS continues to grow in popularity and applications, the field lacks clarity on appropriate stimulation parameters, despite their importance for safe and efficacious neuromodulation. This study aims to review the parameters used in various treatment applications while also providing a concise overview of the mechanisms underlying GVS thereby offering essential context and justification for the chosen parameters. We performed a literature search on the PubMed and Embase databases for clinical trials including the term “galvanic vestibular stimulation.” After removing duplicates, secondary analyses, and studies that did not use GVS for therapeutic purposes, we were left with 53 independent studies. We extracted the stimulation parameters used in each study and report them here. The results of this review suggest that while some stimulation parameters are relatively standardized for specific treatment indications, others lack universally accepted guidelines as the field of GVS continues to evolve. Based on our findings, we recommend that future GVS research include at least one sham condition, the use of individualized current intensity, and the comparison of multiple GVS parameters within the same trial.

## 1 Introduction

The vestibular nerve transmits self-motion and orientation information from the vestibular end organs (the semicircular canals, saccule, and utricle) to the brainstem, cerebellum, and cerebral cortices. This transmission is crucial for oculomotor and postural control, as well as spatial orientation. Additionally, branches of the vestibular nerve project to the mastoid region behind the ear. It is possible to stimulate the vestibular system by applying a low-intensity electric current to the vestibular nerve. This is typically accomplished by placing electrodes on the mastoids and delivering transcutaneous current to local vestibular afferents. This noninvasive form of stimulation is known as Galvanic Vestibular Stimulation (GVS), named after Luigi Galvani who used electric current to evoke frog muscle contractions (Galvani, 1953; Dlugaiczyk et al., 2019). It has been used for its various diagnostic and therapeutic capabilities as well as to investigate vestibular physiology. For example, it can induce a complex whole-body response of oculomotor and balance reflexes, which vary greatly depending on the waveform and other stimulation parameters.

While much of the research on GVS in humans has been restricted to non-invasive methods due to ethical and practical considerations, substantial invasive studies on animals have provided deeper insights into the mechanistic effects of GVS. Johann Purkinje first noted in 1820 that passing electric current through the human head led to balance and equilibrium disturbances (Purkinje, 1820). Later, Josef Breuer attributed these effects to the galvanic stimulation of the vestibular system, observing distinct head movements resulting from the stimulation of individual semicircular canals (SCCs) in birds (Breuer, 1874). Many years later, Chapman et al. (2019) applied targeted GVS to rat SCC nerves and found higher concentrations of c-Fos protein in the contralateral medial vestibular nucleus. These findings by Breuer and Chapman et al. suggest that GVS stimulates the SCC rather than the otoliths. On the other hand, Holstein et al. (2012) applied low-frequency sinusoidal GVS (0.02–0.04 Hz) to rats and found c-Fos protein in the nuclei of otolith-driven neurons, indicating that GVS serves as an otolithic stimulus. To resolve this inconsistency, Kwan et al. (2019) conducted a study in which they inserted recording electrodes into the vestibular nerves of rhesus macaques to measure the activity of individual afferents. Their findings demonstrated that both otolith and SCC afferents are activated by sinusoidal GVS. These results suggest that GVS does not replicate natural head motion as closely as may have been previously assumed, as regular head rotations typically do not stimulate both otoliths and SCC simultaneously.

Nguyen et al. (2021) investigated the effects of GVS on mice following unilateral labyrinthectomy. They found that 1 Hz sinusoidal GVS at 0.1 mA accelerated the recovery of spatial memory and locomotor function in these mice, indicating a potential therapeutic benefit of GVS in vestibular dysfunction. In another study, Sabzevar et al. (2023) applied GVS to rats in direct current (DC) pulses at the minimum intensity that induced eye movement and discovered electrophysiological signals in the tail of the striatum, a recently discovered sensory region, suggesting that vestibular projections also extend into this area. These studies highlight the potential of GVS to influence not only vestibular functions, but also broader neural circuits involved in spatial memory and sensory processing.

The complexity of vestibular signaling and the effects of GVS contribute to significant variability in observed outcomes, which depend on factors such as waveform, anatomical location of stimulation, and other selected parameters.

The mechanisms of suprathreshold and subthreshold are presumably different. Suprathreshold GVS, delivered in either DC or sine waveform, modulates the firing rates of vestibular afferent neurons in a manner that is directly proportional to the applied current, as demonstrated in neural recordings from non-human primates (Kwan et al., 2019; Forbes et al., 2023). This neural activation is subsequently transmitted to the vestibular nuclei in the brainstem, where it mediates a complex whole-body response involving balance reflexes. These reflexes are influenced by various factors, including the head and body’s orientation and position, the specific task being performed, and the sensory input received from other sensory modalities (Fitzpatrick and Day, 2004). In response to this perception, oculomotor and postural responses are triggered directed toward the anodal current (Fitzpatrick and Day, 2004).

Conversely, subthreshold GVS when delivered as a noisy waveform (nGVS) is thought to act by inciting stochastic resonance, increasing the discharge and resting state activity of vestibular afferents, thereby improving neural activation “information transfer” even at intensities that do not cause gross motor activity (Wuehr et al., 2016b). Behavioral responses have been observed that are consistent with this hypothesized mechanism, such as reduced vestibular perceptual thresholds with moderate amplitudes of nGVS (Galvan-Garza et al., 2018), and improvements in balance in both healthy (e.g., Keywan et al., 2018) and diseased populations (e.g., Fujimoto et al., 2018). However, to our knowledge, no studies have quantified vestibular afferent neuron firing rates during nGVS application in non-human primates, as has been done with suprathreshold DC or sine GVS (Kwan et al., 2019; Forbes et al., 2023).

Electrode positioning strongly impacts the induced current flow pattern and electric field at the vestibular organs of interest (Thomas et al., 2020; Truong et al., 2024). This consideration is critical as current flow is believed to modulate the firing rate of vestibular hair cells that is ultimately relayed to the vestibular nuclei in the brainstem (Wardman and Fitzpatrick, 2002). Functional MRI has revealed that bilateral GVS stimulates the temporoparietal junction, central sulcus, anterior interior intraparietal sulcus, and premotor regions of the frontal lobe (Lobel et al., 1999).

Consequently, it is crucial to establish stimulation parameters tailored to the desired application to ensure accuracy and reproducibility. To identify the optimal waveform parameters, we conducted a systematic review of clinical trials on GVS according to the PRISMA guidelines (Page et al., 2021) and extracted stimulation parameters relevant to each application. While GVS is employed both as a diagnostic tool (Dlugaiczyk et al., 2019) and as a method to enhance or modulate balance in healthy individuals (e.g., Wuehr et al., 2016a; Brunyé et al., 2024), this review specifically concentrates on its therapeutic applications. Additionally, although other forms of vestibular stimulation, such as thermal (e.g., caloric) and invasive techniques, are worthy of consideration, our focus is on conventional, non-invasive GVS.

## 2 Methods

We conducted a literature search of the PubMed and Embase databases for clinical trials with the keyword “galvanic vestibular stimulation” up to July 1, 2024. We included research articles, letters, and case studies, but excluded literature reviews and abstracts. A research team member screened each result and removed duplicates, studies that used GVS solely for diagnostic or screening purposes, and studies that applied GVS simply to enhance functions in healthy subjects rather than to treat a specific symptom or disorder. We segmented the remaining results into three groups: (1) GVS for treating impaired postural control, (2) GVS for post-stroke patients, and (3) GVS for other clinical applications. We extracted the stimulation parameters of each study as well as the study design and number of diseased patients in each arm. We only report that a study was double-blind when the authors mention that the investigators were blinded to the treatment conditions. Otherwise, we assume that blinded studies were only single-blind.

The stimulation parameters included electrode montage, waveform, frequency, intensity, sham procedure, stimulation length, number of sessions, and inter-session interval as follows:

### 2.1 Electrode montage

**Figure 1:**
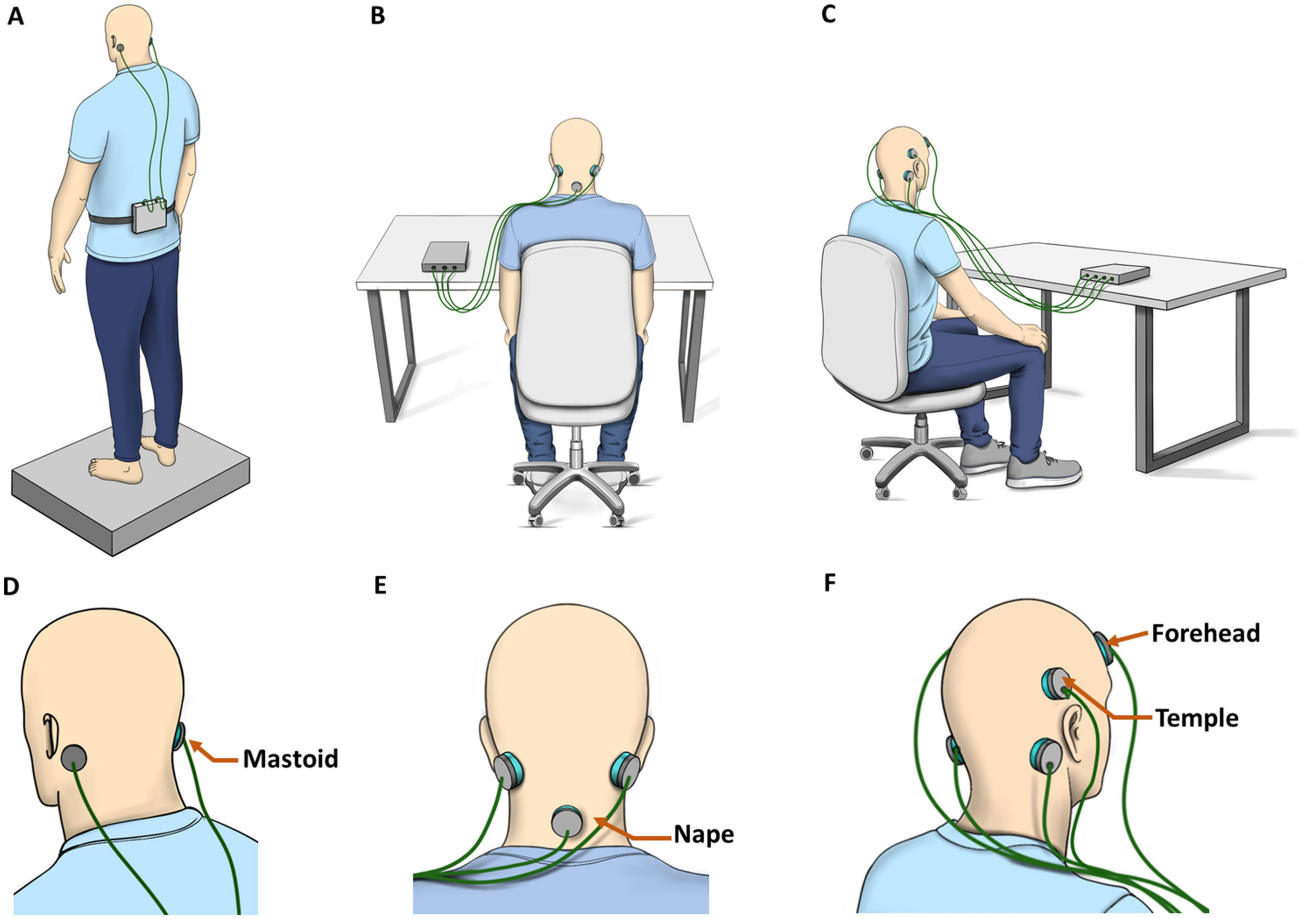
GVS administration and electrode montage. **(A)** Bilateral-Bipolar is the most common. It is often delivered while the subject stands on a force plate. **(B,C)** Multichannel setups are tested more frequently for motion sickness and cybersickness applications by placing additional electrodes on the back of the neck, temples, or forehead. **(D–F)** Corresponding expanded images indicating electrode locations tested in the literature.

Electrode montage describes the anatomical positions of the electrodes as well as the polarity pattern of the electrodes. Most commonly, GVS is delivered through one electrode on each mastoid, a montage known as “bilateral-bipolar” (Figure 1A). Some studies have examined alternative electrode montages or additional electrodes at locations such as the forehead or temples (e.g., Cevette et al., 2012; Aoyama et al., 2017; Figures 1B,C).

### 2.2 Waveform

**Figure 2:**
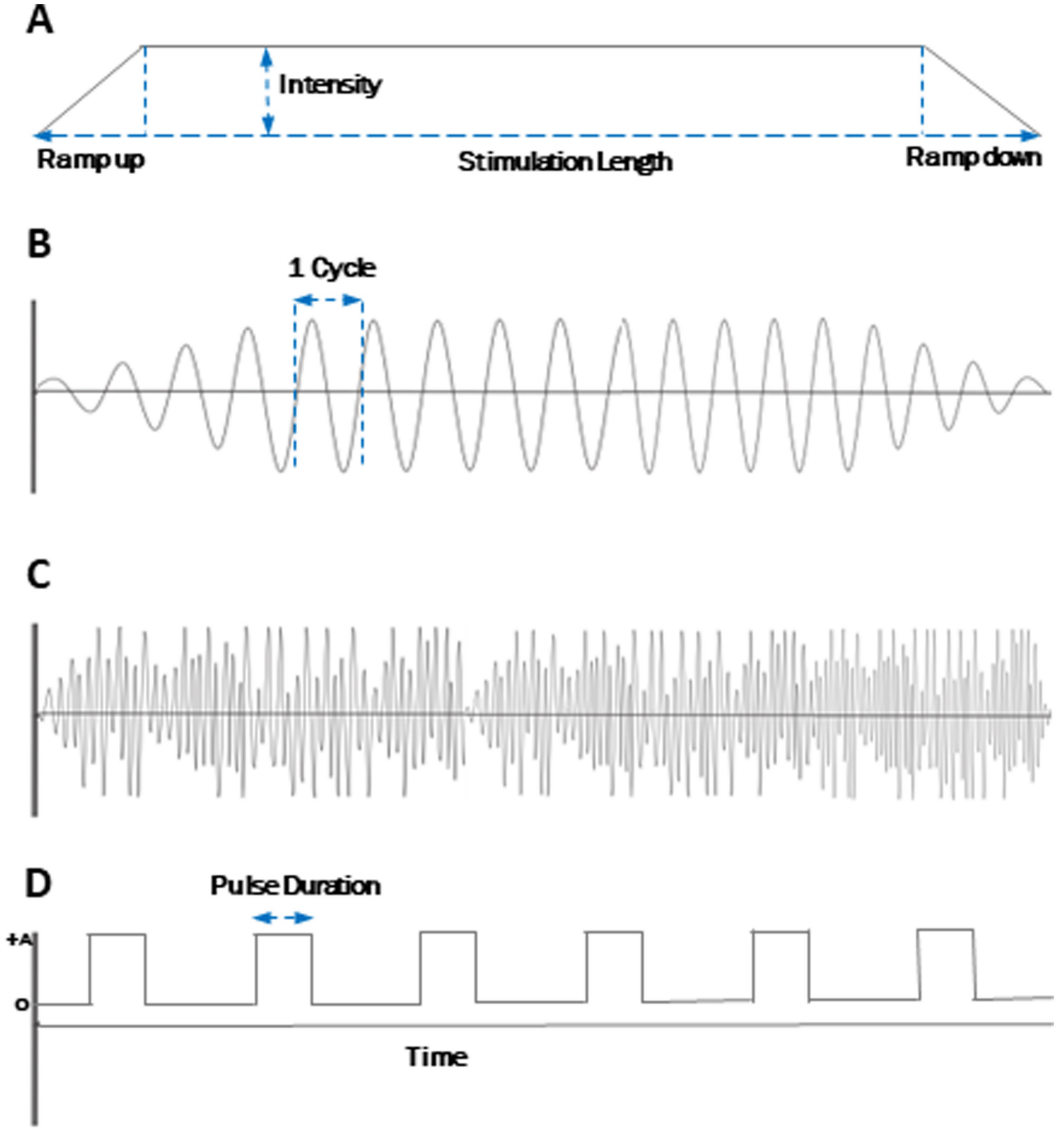
Electrical waveform parameters for GVS. **(A)** Direct Current (DC). **(B)** Sinusoidal. **(C)** Noisy. **(D)** DC pulses. Frequency is the number of cycles per second. Stimulation is generally ramped up and ramped down at the beginning and the end of the session to prevent cutaneous discomfort. An exemplary noise waveform is shown here and does not specifically differentiate from different implementations (white noise, random noise, etc.) Similarly, the DC pulse shown is exemplary and may or may not be offset from the 0 line.

Waveform is the shape of the electric current function. It is independent of amplitude or time. Different waveforms of GVS have different physiological effects and are therefore used for different applications. The most commonly used GVS waveforms are DC, sinusoidal, and stochastic noisy white noise. Figure 2 presents visual representations of these parameters as simplified electrical waveforms.

#### 2.2.1 Direct current

Depending on the polarity and intensity, DC causes an immediate and sustained change in the firing rate of the neurons (Bindman et al., 1964). Sometimes, GVS in a DC waveform is applied as a train of short pulses. When this is the case, we mention the time duration of each pulse (i.e., pulse width), if reported by the authors. Figure 2D illustrates DC pulses and the definition of pulse duration.

#### 2.2.2 Sinusoidal

Sinusoidal GVS induces sensations and vestibulo-ocular reflexes that resemble those elicited by head rotations (Kim et al., 2011; Gensberger et al., 2016). However, GVS does not perfectly replicate natural head movements, as it likely stimulates both the SCC and the otolith organs (Kwan et al., 2019), whereas natural head motion typically does not. In rare instances, a combination of sine waves of different frequencies are combined into a single stimulation waveform, known as multisine (Kazemi et al., 2021; Lee et al., 2021a; Liu et al., 2021).

#### 2.2.3 Noisy

Producing stochastic resonance by applying band-limited nGVS is assumed to reduce the firing threshold of vestibular irregular afferent neurons boosting weak physiological signals from the vestibular apparatus (Nooristani et al., 2021; Eder et al., 2022; Wuehr et al., 2023).

### 2.3 Frequency

Frequency is a measure of the rate of oscillatory fluctuations of a waveform. While DC frequency is zero, sinusoidal current is typically applied at a specified frequency (i.e., the sine wave frequency) and nGVS is typically delivered within a defined frequency spectrum that encompasses all frequencies within the specified range (e.g., a bandwidth of 0.01 to 2.0 Hz). The electric current delivered varies across frequencies within this range.

### 2.4 Intensity

Current intensity is a measure of the amplitude, or strength, of the electric current. Given the weak nature of current applied in GVS, this is typically reported in milliampere (mA) units. While some studies have used a fixed intensity across all subjects, *individualized* current intensity (determined pre-trial) is also used. Sometimes, this was done to ensure that the current intensity was below the subject’s sensory threshold (either cutaneous or vestibular) so as not to compromise subject blinding. In other studies, it was done to determine the highest or most optimal current level based on previous literature without surpassing limits of safety and tolerability. We also report the mean and standard deviation of the threshold when provided by the paper. For sine and noisy waveforms where the intensity fluctuates, we report the maximum intensity.

### 2.5 Sham

Some studies compare the effects of real GVS to sham (i.e., placebo) stimulation. We report whether studies used a sham comparison and how it was implemented.

### 2.6 Stimulation length

Stimulation length is the duration that stimulation is delivered during a single session. A longer stimulation length is generally believed to have a greater effect. We note that some studies only report that stimulation was delivered throughout the duration of a task, but do not report the time duration of the task. In these cases, we report that stimulation length was not specified.

### 2.7 Number of sessions

The number of sessions is the total number of stimulation sessions delivered. The importance of the number of sessions is that it considers the effect of cumulative dosage (Thompson et al., 2021).

### 2.8 Inter-session interval

Inter-session interval is the time duration between stimulation sessions. Sometimes stimulation was delivered while subjects performed tasks where there was a break between each task to avoid potential after-effects. In may be unclear how long the breaks were between stimulations. It then becomes important for the reader to carefully track the full methodology to retrieve the exact timing. In studies that involved multiple successive trials, some of the trials could have active stimulation while others were shams. As a result, inter-session interval is sometimes not straightforward to comprehend and could imply the time duration between trains of stimulation pulses or the interval between a group of trials. When the session is repeated multiple times in a day (e.g., Fujimoto et al., 2016), this ambiguity may lead to further confusion. In this study, we sought to make this distinction clear as well as the specified number of hours and/or days between stimulation sessions. In summary, besides the ambiguity in interpreting length of stimulation session and inter-session intervals in some studies, other parameters are reported in precise terms. The parameters we listed above serve as a basis to systematically compare across the studies included in this review.

## 3 Results

**Figure 3:**
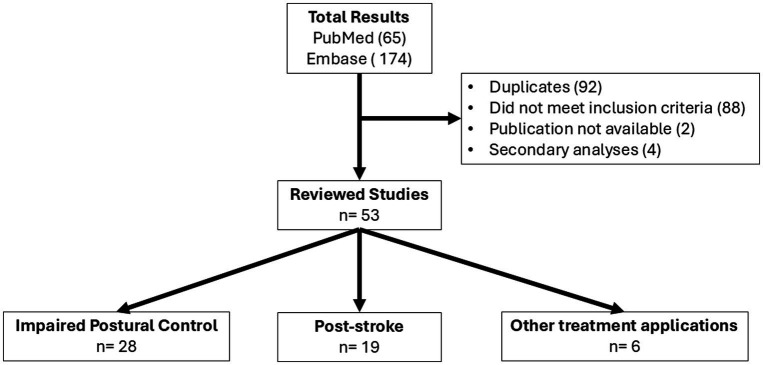
Flow of study selection procedure.

Our search returned a total of 239 results. We then removed 92 duplicates, 88 articles that did not meet the inclusion criteria, and 2 papers that were not available (Figure 3). Among the remaining 57 publications, four of them were secondary analyses of other studies. This left us with 53 independent studies discussed below.

### 3.1 GVS for impaired postural control

Impaired upright postural control often arises from disruptions in the sensory information that governs motor skills, as well as the body’s awareness of its position in relation to its surroundings and spatial orientation. Such disturbances can be attributed to disorders of the vestibular system, which may result from factors such as head injury, viral infections, and various genetic and environmental influences. Common symptoms of vestibular system pathology include loss of balance, vertigo, dizziness, blurred vision, nausea, lack of coordination, vomiting, unsteady gait, and muscle aches (Thompson and Amedee, 2009; Pérez-Fernández and Ramos-Macías, 2023).

In addition to these factors, aging contributes to progressive functional decline that increasingly compromises balance. The natural loss of muscle mass (sarcopenia), along with neurological and psychiatric disorders, vestibular impairments, certain medications, and cardiovascular disease, can exacerbate balance deficits among older adults. These balance disorders represent a significant risk factor for falls, which are a major public health concern, as they account for the leading cause of accidental death in individuals over the age of 60 (Gaspar and Lapão, 2021).

Impaired postural control can also be the result of neurodegenerative disorders such as Parkinson’s Disease (PD), an incurable chronic neurological disorder characterized by the degeneration of nerve cells in the substantia nigra. Symptoms of PD include postural imbalance, tremors, rigidity, bradykinesia (slowness of movement), and stiffness of limbs (Hodgson et al., 2021).

The peripheral vestibular system, along with its associated brain regions, plays a critical role in the collection and integration of vestibular signals. Consequently, modulation of the vestibular system presents a promising approach for addressing postural control deficits in humans. GVS is particularly advantageous due to its minimal and transient side effects, making it suitable for repetitive treatments and compatible with concurrent tasks, such as rehabilitation therapy (Volkening et al., 2016). Moreover, recent studies have revealed that GVS delivered in a noisy waveform can induce neurochemical changes in key regions, including the substantia nigra and the parvocellular medial vestibular nucleus, both of which are implicated in PD (Lee et al., 2021b). This suggests that GVS may not only aid in improving postural control but also influence underlying neurophysiological mechanisms related to PD.

Various stimulation parameters of GVS have been used to improve impaired postural control in persons with vestibular disorders, elderly adults, and in patients with neurodegenerative disorders. We present parameters of each study in Table 1. Table 2 serves as a summary providing the most commonly utilized parameters across GVS studies for impaired postural control as well as the full range of parameters.

**Table 1:** GVS stimulation parameters for improving postural control in various populations and reported clinical outcomes.

Study	Electrode montage	Waveform	Frequency (Hz)	Current intensity (mA; mean ± SD)	Length of stimulation session	Number of sessions	Inter-session/inter-test day interval	Study design and size	Sham procedure	Clinical outcomes
Bilateral Vestibulopathy										
Iwasaki et al. (2014)	Bilateral mastoids	Noisy	0.02–10	0.1–1	30 s	6 (1 of each intensity level)	3 min	Single blind, crossover *n* = 11	0 mA	Improvement in sway velocity, envelopment area, and COP RMS
Wuehr et al. (2016b)	Bilateral mastoids	Noisy	0–30	80% of cutaneous sensory threshold (0.3815 ± 0.0383)	min	6	3 min	Single-blind, crossover. *n* = 13	0 mA	GVS during walking improved variability and bilateral coordination gait measures, but not spatiotemporal gait measures
Fujimoto et al. (2018)	Bilateral mastoids	Noisy	0.02–10	Greatest postural control (0.46 ± 0.06)	30 min	1^*^	-	Open-label. Active *n* = 13	-	Postural balance was significantly improved for 6 h. post-stimulation measured by COP movement and subjective rating
Iwasaki et al. (2018)	Bilateral mastoids	Noisy	0.02–10	0.1–1	15 min	10	≥ 3 min	Single-blind, crossover. *n* = 12	0 mA	Significant improvement in gait velocity, stride length, and stride time.
Ko et al. (2020)	Bilateral mastoids	Noisy	0.02–10	Greatest postural control	6 min	1	-	Open-label. Active *n* = 7	-	Changes in EEG activity and significant improvement in RMS of COP and postural control
Sprenger et al. (2020)	Bilateral mastoids	Noisy or Sine^**^	Noisy: 0.2–20. Sine: 1	0.5, 1.5, or 80% vestibular threshold (0.81 ± 0.06)	12 s	36	14 s	Single-blind, crossover. *n* = 30	Ramp up and ramp down followed by 0 mA	No consistent balance stabilizing effect of GVS in Bilateral Vestibulopathy patients
Fujimoto et al. (2021)	Bilateral mastoids	Noisy	0.02–10	Greatest postural control	30 min	2	14 days	Open-label. Active *n* = 13	-	Some subjects experienced a minimally important difference in symptoms
Eder et al. (2022)	Bilateral mastoids	Noisy	0–30	Greatest postural control (0.33 ± 0.2)	30 min	6	3 times per week	Double-blind. Active *n* = 12; sham *n* = 11	0 mA	No significant improvement in Timed Up and Go Test or Functional Gait Assessment in VRT + GVS compared to VRT alone
Wuehr et al. (2023), Wuehr et al. (2024a)	Bilateral mastoids	Noisy	0–30	0.1–0.7	30 s	8	unspecified	Single-blind, crossover. *n* = 11	0 mA	nGVS resulted in lowered perceptual thresholds in most patients. Optimal intensity was approximately 0.4 mA
Unilateral peripheral vestibular syndromes										
Carmona et al. (2011) ^***^	Bilateral mastoids	DC (cathode on the side of vestibular sway)	-	2	1 min	5	1 session per week	Open-label. Active *n* = 10, control *n* = 40	-	A significantly higher percentage of patients attained complete improvement in the GVS + VRT group than in the VRT group.
Carmona et al. (2011) ^***^	Bilateral mastoids	DC (cathode on the side of vestibular sway)	-	2	1 min	2 or 5	1 session per week	Single-blind. 5 sessions *n* = 15; 2 sessions *n* = 5	Electrodes on clavicle	Ideal response was found after 3 sessions of GVS
Unilateral or Bilateral Vestibulopathy or Cerebellar Ataxia										
Nguyen et al. (2023)	Bilateral mastoids	DC, Noisy, Sine	Sinusoidal: 1 Noisy: 0–100	0.4, 0.8, or 1.2	5 or 30 min	12–18	30 min	Open-label. *n* = 31	-	Optimal parameters varied depending on the treatment population.
Dizziness and Vertigo										
Ceylan et al. (2020)	Bilateral mastoids	0.5 ms DC pulses	100	3–4	10 min	6	Once a week	Open-label. Active *n* = 42; control *n* = 35	-	Significant improvement in the GVS + VRT group compared to VRT alone in unilateral weakness and some measures of balance and stability.
Elderly Adults										
Fujimoto et al. (2016)	Bilateral mastoids	Noisy	0.02–10	Greatest postural control (0.18 ± 0.01)	Day 1: 30 min; Day 2: 3 h	Day 1: 2 Day 2: 1	Day 1: 4- Day 1 and 2 separated by 7 days	Open-label. Active *n* = 20	-	Postural stability was significantly improved for >2 h. after stimulation ended on both Day 1 and Day 2. Session 2 resulted in even greater improvement than Session 1, indicating a cumulative effect.
Ankit et al. (2020)	Bilateral mastoids	Noisy	unspecified	unspecified	20 min	18	3 times per week	Single-blind. Active *n* = 50; sham *n* = 50; control *n* = 50	0 mA	Active group had a significant improvement in overall stability index and Timed up and go test compared to sham
Nooristani et al. (2021)	Bilateral mastoids	Noisy	0.1–640	0.24 mA	30 min	1	-	Single-blind. Active *n* = 24; sham *n* = 12	Ramping up and down at the beginning and end with 0 mA in between	Significant improvement in sway velocity and path length.
Parkinson’s Disease										
Lee et al. (2015)	Bilateral mastoids	Noisy	0.1–10	90% of cutaneous sensory threshold (0.19–0.9)	90 s	8	30 s	Single-blind, crossover. *n* = 12	0 mA	GVS improved performance on a visuomotor tracking task
Okada et al. (2015)	Bilateral mastoids and trapezius muscles	DC (cathodes on mastoids, anodes on trapezius muscles)	-	0.7	20 min	1	-	Single-blind, crossover. *n* = 7	Ramped up to 0.7 mA, then down to 0 mA	Significant decrease in anterior bending angles in both the eyes-closed and eyes-open tests
Cai et al. (2018)	Bilateral mastoids	Noisy then Sinusoidal	Noisy: 0.1–1; Sine: 1	90% of cutaneous sensory threshold	5 min	1 of each waveform	2 min	Open-label, active *n* = 30	-	Both stimulation waveforms significantly increased the overall PPN connectivity magnitude, but connectivity of specific regions varied between waveforms
Khoshnam et al. (2018)	Bilateral mastoids and forearms	DC (cathodes on mastoids, anodes on forearms)	-	200% of cutaneous sensory threshold (0.12–1.5)	20 s for one task, unspecified length for another task	6	3 min	Single-blind, crossover *n* = 11	0 mA	Statistically significant improvement in the Instrumented Timed Up and Go test and finger tapping task
Tran et al. (2018)	Bilateral mastoids or bilateral mastoids with an additional two electrodes on shoulders	Noisy (monopolar or bipolar)	0.4–30	70% of cutaneous sensory threshold (0.22 ± 0.02)	60 s	4	60 s	Single-blind, crossover. *n* = 13	-	Measurable postural changes. Stimulation did not change sway amplitude, but monopolar stimulation mildly reduced sway frequency.
Kazemi et al. (2021)	Bilateral mastoids	Multisine	50–100 or 100–150	Unspecified	90 s	10 per waveform	2 min	Single-blind, crossover. *n* = 18	0 mA	Both 50–100 Hz and 100–150 Hz improved motor vigor
Lee et al. (2021a)	Bilateral mastoids	Noisy or Multisine	4–200 for both Noisy and Multisine	90% of cutaneous sensory threshold (0.45 ± 0.22)	100 s	9	2 min	Single-blind, crossover. *n* = 18	-	Significant improvement in reaction time task for some stimulation frequencies. The optimal stimulus frequency varied between individuals.
Liu et al. (2021)	Bilateral mastoids	Noisy, Sine, and Multisine	0.1–10 for Noisy, 1 for Sine, 70–200 for Multisine	90% of cutaneous sensory threshold	5 min	1 of each waveform	2 min	Single-blind, crossover. *n* = 32	-	Sinusoidal and multisine GVS improved subnetwork interactions more effectively than noisy GVS observed using fMRI
Other Neurodegenerative Disorders										
Yamamoto et al. (2005)	Bilateral mastoids	Noisy	0.01–2.0	60% of sensory threshold (sensory threshold: 0.33 ± 0.20)	24 h	1		Single-blind, crossover. *n* = 19	0 mA	Significant increase in HRV, antipersistence in trunk activity, and decrease in reaction time, indicating improved autonomic and motor responsiveness
Pan et al. (2008)	Bilateral mastoids	Noisy	0.01–2.0	60% of nociceptive threshold (sensory threshold: 0.33 ± 0.20)	24 h	1	-	Double-blind, crossover. *n* = 14	0 mA	GVS decreased akinesia compared to sham indicating improved motor function
Lotfi et al. (2021a)	Bilateral mastoids	Noisy	0–30	90% of cutaneous sensory threshold (0.54 ± 0.40)	30 min	12	Twice a week	Single-blind. GVS *n* = 8; VRT *n* = 8; control *n* = 8	0 mA	No significant difference compared to control in the sensory organization test, Dizziness Handicap Inventory, or Activities-Specific Balance Confidence Scale
Wuehr et al. (2024b)	Bilateral mastoids	Noisy	0–30	0.1–0.7	30 s	8	unspecified	Single-blind, crossover. *n* = 16	0 mA	Half of the subjects exhibited clinically meaningful effects with the greatest response at 0.3 mA

When only 1 stimulation session was performed, we entered ‘-’ in the inter-session column. HRV, Heart Rate Variability; COP, Center of Pressure; VRT, Vestibular Rehabilitation Therapy; PPN, Pedunculopontine Nucleus; RMS, Root Mean Square.

* An additional session was conducted to test reproducibility.

** Sine waveform is assumed. The study only mentions 1 Hz alternating current stimulation.

*** Both experiments are reported in a single paper.

**Table 2:** Most common parameters and range of parameters used for improving postural control.

	Electrode montage	Waveform	Frequency (Hz)	Intensity (mA)	Sham procedure	Length of stimulation session	Number of sessions	Inter-session/inter-test day interval
Most common parameter	Bilateral mastoids	Noisy	No commonly used frequency	Individualized intensity of greatest postural control	0 mA	No commonly used length	1 per waveform	No commonly used interval
Range of parameters	Bilateral mastoids, trapezius muscles, forearms, shoulders, C7 vertebra	Noisy, DC, DC pulses, Sine, Multisine,	0.01–640	0.1–1.5	No sham, 0 mA sham, current delivered to a different anatomical location, ramping up and down at the beginning and end	12 s–24 h.	1–36	14 s–1 week

#### 3.1.1 Electrode montage, waveform, and frequency

For impaired postural control, the objective of GVS is to compensate for dysfunctional vestibular function by enhancing the sensory input to the vestibular system. This is typically achieved by delivering a noisy waveform to the vestibular afferents that project to the mastoid region. This stimulation lowers the neural firing threshold, thereby amplifying weak physiological signals. This method has been applied across various populations with impaired postural control, resulting in notable improvements in autonomic and motor functions in patients with neurodegenerative disorders (Yamamoto et al., 2005; Pan et al., 2008; Khoshnam et al., 2018; Lee et al., 2021a), as well as enhanced postural stability in elderly adults (Fujimoto et al., 2016; Nooristani et al., 2021) and individuals with bilateral vestibulopathy (Iwasaki et al., 2014; Fujimoto et al., 2018; Wuehr et al., 2016b; Iwasaki et al., 2018; Ko et al., 2020; Fujimoto et al., 2021). Notably, Fujimoto et al. (2016) reported that elderly participants experienced improved postural stability several hours after stimulation had ceased. The bilateral-bipolar montage was the predominant configuration used by the majority of studies evaluated in this review, indicating it as the preferred montage choice.

An alternative, less commonly used stimulation setup involves applying DC through two cathodal electrodes placed on the mastoids and two anodal electrodes positioned on the trapezius muscles (Okada et al., 2015) or forearms (Khoshnam et al., 2018). This electrode configuration allows for the stimulation of vestibular nerves on both sides simultaneously with anodal current, which is not feasible with bilateral-bipolar stimulation. Reports indicate that this method can improve anterior bending posture and other motor symptoms in patients with PD (Okada et al., 2015; Khoshnam et al., 2018).

When addressing peripheral vestibular syndromes, the aim shifts to stimulating the very sensory system that is known to be damaged. In cases where the peripheral disorder is unilateral, DC is delivered with the anodal electrode on the contralateral side of the impairment to counteract the patient’s mediolateral sway. Consequently, Carmona et al. (2011) applied DC stimulation in conjunction with vestibular rehabilitation therapy (VRT) to patients with unilateral peripheral vestibular syndromes, positioning the anodal electrode on the contralateral side of the impairment.

Frequency is not applicable for DC stimulation but is a major consideration in noisy GVS protocols. There is variability, however, with respect to the actual administration of the noisy waveforms, with spectral content varying from close to 0 Hz to low 10s or sometimes even up to 640 Hz. As discussed in a review by McLaren et al. (2023) the choices of different frequencies are supported by various rationales related to the physiology of vestibular organs or the central nervous system. Body sway typically occurs within a frequency range of 0.02–10 Hz (Assländer et al., 2021; Lajoie et al., 2021), whereas normal head motion during walking is found in the 0–2 Hz range (Hirasaki et al., 1999). Conversely, a frequency range of 0–30 Hz has been identified as effective for stimulating vestibular hair cells and is regarded as the natural frequency of the vestibular system (Mulavara et al., 2011; Goel et al., 2015; Mulavara et al., 2015; Dlugaiczyk et al., 2019). Alternatively, some studies have used a frequency range of 0 to 640 Hz, as this range has been shown to activate cortical neurons (Terney et al., 2008), suggesting its potential for effective GVS outcomes. Also, due to this ability to stimulate cortical activity, this frequency range is commonly employed in tES devices, which are widely utilized in GVS research. Therefore, in these instances, the choice of frequency band may simply be due to the available setting on the stimulator.

Given that most studies report beneficial outcomes, it may be inferred that precise tuning of frequency content is less critical than otherwise assumed. However, the broad frequency ranges often employed might result in suboptimal application, suggesting that effects could be further enhanced with more targeted and precise frequency settings.

#### 3.1.2 Intensity and sham control

Some investigators have delivered stimulation just below the subject’s sensory threshold (Lee et al., 2021a; Liu et al., 2021; Lotfi et al., 2021a), to enable blinding for controlled studies. This way the subjects would not know whether they received active or sham stimulation. Other studies delivered stimulation at the subject’s optimal intensity to maximize stimulation benefit (Fujimoto et al., 2016; Fujimoto et al., 2018; Ko et al., 2020). For example, Fujimoto et al. (2016) used a dosage titration technique before each experimental session by measuring three parameters during various current intensities: the mean velocity, envelopment area, and the root mean square of the center of pressure (COP) movement in the XY plane. They identified the optimal intensity of noisy GVS as the intensity which improved all these parameters simultaneously during the stimulus compared with baseline. Notably though, Eder et al. (2022) performed the same optimization technique on the same population and combined the stimulation with vestibular rehabilitation therapy (VRT). Results showed no additional effects of GVS compared to patients who received VRT alone. The authors suggest that the lack of combined effect may be due to the difference in mechanisms as low intensity GVS affects sub-threshold vestibular cues, while the VRT protocol employed in the study focused on fast head kinematics. Conversely, the aforementioned study by Carmona et al. (2011) found that patients with unilateral peripheral vestibular syndromes undergoing VRT and GVS at 2 mA intensity were more likely to recover than subjects who received VRT alone. The difference in clinical outcomes between Carmona et al. and Eder et al. may suggest that a VRT-GVS synergy is present when suprathreshold intensity is used, as opposed to when subthreshold GVS is used. Alternatively, the difference in clinical outcomes between these two studies may have been due to differences in subject population (unilateral vs. bilateral vestibular pathology) or waveform (noisy vs. DC).

Other studies delivered stimulation uniformly to all subjects at a single intensity, such as 0.4 mA, (e.g., Inukai et al., 2018) or a range of intensities, such as 0.1–0.7 mA, to test the effects of GVS at different intensities (e.g., Wuehr et al., 2023). In studies that delivered supersensory stimulation, a topical anesthetic can be applied to minimize sensation so as to improve subject blinding (Sprenger et al., 2020).

While some GVS studies were open-label without a sham control, others used a study arm that received 0 mA throughout (e.g., Kazemi et al., 2021) or a brief stimulation period where current was delivered at the beginning of the session and then ramped down without informing the subject (Okada et al., 2015; Sprenger et al., 2020; Nooristani et al., 2021), a technique often used as sham in studies on transcranial electrical stimulation (tES). Studies focusing solely on dose optimization (e.g., Nguyen et al., 2023) were less likely to utilize a sham control, as the different doses themselves served as an internal control for comparison. We note the non-ideal sham strategy in Carmona et al., 2011, where they applied stimulation to the mastoids in the active arm and off-target (clavicle) in the sham arm. While the study may have assessed blinding efficacy, it was not reported. Similarly, Khoshnam et al., 2018 reported that the sham condition was administered as “GVS off.” Given active arm delivered stimulation above the cutaneous threshold, it is unclear how blinding efficacy was maintained.

#### 3.1.3 Stimulation sessions: length, number, and interval

Most studies have primarily examined the effects of a single galvanic vestibular stimulation (GVS) session (Yamamoto et al., 2005; Pan et al., 2008; Okada et al., 2015) or a single session of each waveform (Iwasaki et al., 2014; Liu et al., 2021). A limited number of studies have explored the cumulative effects of multiple sessions, particularly in conjunction with VRT (Carmona et al., 2011; Ceylan et al., 2020).

The duration of GVS sessions varied considerably among studies, influenced by whether the aim was to assess the immediate effects of stimulation—wherein brief stimulation suffices for testing—or to evaluate the prolonged effects resulting from extended stimulation. For instance, Inukai et al. (2018) administered only 30 s of stimulation and measured improvements in postural control among elderly adults during the stimulation. In contrast, Ankit et al. (2020) implemented 20-min sessions for the same demographic and analyzed the effects of prolonged stimulation. Studies investigating extended stimulation typically limited the duration to 20 or 30 min, adhering to safety protocols established for transcranial direct current stimulation (tDCS; Woods et al., 2016; Bikson et al., 2016).

To prevent carryover effects between sessions to effectively assess each waveform independently, studies implemented intervals between different stimulation waveforms. The intervals varied significantly, with some studies using 2-min breaks (Cai et al., 2018; Liu et al., 2021; Lee et al., 2021a) and others opting for longer breaks such as a 30-min interval (Nguyen et al., 2023). When investigating cumulative effects through repeated doses, studies typically adopted regimens that included treatments administered once, twice, or three times per week (Carmona et al., 2011; Ankit et al., 2020; Ceylan et al., 2020; Lotfi et al., 2021a; Eder et al., 2022).

### 3.2 GVS for spatial orientation in stroke patients

Recent medical advancements have increased the percentage of stroke survivors. However, the survivors are left to cope with the psychological, functional, physical, and social effects of stroke. A common lasting effect of both ischemic and hemorrhagic stroke is an impairment in the ability to integrate visual, somatosensory, and vestibular information (Saeys et al., 2018; Barra et al., 2010; Utz et al., 2011a). This often results in a misperception of verticality as indicated by deviations in the patients’ subjective visual vertical (SVV; Utz et al., 2011b; Oppenländer et al., 2015a). The inability to integrate these channels also shifts the center of gravity towards the paretic side, impairing postural balance to the extent that sitting or standing can become impossible (Volkening et al., 2016), aside from postural difficulties arising from muscle weakness. Patients will also often actively push with the non-affected extremities towards the paretic side and exhibit resistance to passive correction, a condition called pusher behavior (Volkening et al., 2016). Difficulty in integrating various sensory channels post-stroke also often results in spatial neglect (Barra et al., 2010; Utz et al., 2011b), which is the impairment in detecting or responding to sensory stimuli in contralesional space (Heilman et al., 2000). Patients often show left-sided spatial neglect after damage to the right hemisphere (Volkening et al., 2016). When spatial neglect is present, a deviation of the egocentric reference frame towards ipsilesional space is experienced (Karnath, 1994; Vallar, 1997).

Through GVS activation of the vestibular cortex, the subjective vertical inclines to the anodal side, shifting the center of gravity to the anodal side as well (Oppenländer et al., 2015a). As such GVS has been investigated as a treatment mechanism for improving these symptoms in stroke patients. The parameters used are enumerated in Table 3. Table 4 serves as a summary providing the most commonly utilized parameters across GVS studies for post-stroke patients as well as the full range of parameters.

**Table 3:** GVS stimulation parameters for post-stroke patients and reported clinical outcomes.

Study	Electrode montage	Waveform	Frequency (Hz)	Current intensity (mA; mean ± SD)	Length of stimulation session	Number of sessions	Inter-session/inter-test day interval	Study Design and Size	Sham procedure	Clinical outcome
Right-hemispheric Stroke										
Rorsman et al. (1999)	Bilateral mastoids	DC (anodal-left)	-	Just below sensory threshold (median-1.15)	Throughout duration of test (unspecified length of time)	1–2	1 day	Single-blind, crossover. *n* = 14	0 mA	Significantly larger effect in line crossing task, but not in star cancelation task.
Wilkinson et al. (2005)	Bilateral mastoids	DC (anodal-right or anodal-left)	-	90% cutaneous sensory threshold (mean: 1.2)	Not specified	1 of each waveform	15 min	Single-blind, crossover. *n* = 1	0 mA	Face recognition improved after two GVS sessions
Saj et al. (2006)	Bilateral mastoids	DC (anodal-left)	-	1.5	Throughout duration of test (unspecified length of time)	1	-	Single-blind, crossover. *n* = 12	0 mA	GVS influenced SVV, especially when spatial neglect was present
Wilkinson et al. (2009)	Bilateral mastoids	DC (anodal-left)	-	1	30 min	5	1 day	Open-label, crossover *n* = 1	-	No deterioration in global function observed
Wilkinson et al. (2010)	Bilateral mastoids	DC (anodal-right or anodal-left)	-	90% sensory threshold (mean 1.1)	Approximately 6 min	7	5 min or 2 weeks	Single-blind, crossover. *n* = 1	0 mA	Both waveforms improved accuracy on the figure copy task
Kerkhoff et al. (2011)	Bilateral mastoids	DC (anodal-right or anodal-left)	-	0.5–0.6	20 min	1 of each waveform	1 week	Single-blind, crossover. *n* = 2	ramp up, ramp down and then 0 mA	Chronic tactile extinction improved depending on polarity
Utz et al. (2011a), Utz et al. (2011b)	Bilateral mastoids	DC (anodal-right or anodal-left)	-	0.1 below sensory threshold (mean 0.6) or 1.5	15–20 min	1 of each waveform	1 day	Single-blind, crossover. 1.5 mA *n* = 19; subsensory *n* = 36	ramp up, ramp down and then 0 mA	1.5 mA anodal-left GVS reduced pathological rightward shift in horizontal line bisection. Both stimulation intensities induced few and mild adverse effects
Schmidt et al. (2013a), Schmidt et al. (2013b)	Bilateral mastoids	DC (anodal-right or anodal-left)	-	0.1 mA below cutaneous sensory threshold (mean: 0.7 or 0.6)	Throughout duration of test (unspecified length of time)	1 of each waveform	48 h	Single-blind. Active *n* = 6; sham *n* = 6	0 mA	Both waveforms improved tactile identification compared to sham. GVS also improved arm position sense during stimulation and even 20 min after stimulation ended
Zubko et al. (2013)	Bilateral mastoids	DC (anodal-left)	-	90% of cutaneous sensory threshold (1 or 1.5)	20 min	5	1 day	Open-label, crossover *n* = 2	-	Both subjects improved on the Behavioral Inattention Test up to 3 days post-stimulation
Nakamura et al. (2014)	Bilateral mastoids	DC (anodal-right)	-	0.3–2	20 min	10	1 day	Open-label, crossover. *n* = 2	-	There was an improvement in SCP scores
Ruet et al. (2014)	Bilateral mastoids	DC (anodal-right or anodal-left)		1.5	20 min	1 of each waveform	48 h	Single-blind, crossover. *n* = 4	ramp up, ramp down and then 0 mA	No significant difference in line bisection and star cancelation following GVS relative to sham
Wilkinson et al. (2014)	Bilateral mastoids	Noisy (non-zero mean, anodal-left)	Not specified	0.5–1.5 (mean: 1)	25 min	1,5 or 10	1 day	Double-blind. One session *n* = 17; five sessions *n* = 18; 10 sessions *n* = 17	0 mA	Statistically significant improvement in all three treatment arms between baseline and 4 weeks post-GVS on BIT
Nakamura et al. (2015)	Bilateral mastoids	DC (anodal-right or anodal-left)	-	Below sensory threshold (0.4–2.0 mA)	20 min	1 of each waveform	48 h	Single-blind, crossover. *n* = 7	0 mA	Left-cathodal GVS but not right-cathodal GVS significantly improved task performance. Twenty minutes of stimulation caused a greater improvement than 10 min.
Oppenländer et al. (2015a); Oppenländer et al. (2015b)	Bilateral mastoids	DC (anodal-right or anodal-left)	-	Just below cutaneous sensory threshold (mean- 0.7, range: 0.4–1.5)	20 min	1 of each waveform	2 days	Single-blind, crossover. *n* = 24	ramp up, ramp down and then 0 mA	Left-cathodal GVS improved SVV, STV, line bisection, and figure copying. Right-cathodal GVS improved figure copying and digit cancellation tasks, with a small improvement in SVV and STV
Stroke										
Krewer et al. (2013)	Bilateral mastoids	DC (anodal- ipsilesional mastoid)	-	Vestibular sensory threshold	20 min	1	-	Double-blind, crossover. *n* = 25	-	GVS slightly improved pusher behavior, but the effect did not reach significance
Bonan et al. (2016)	Bilateral mastoids	DC (anodal-right or anodal-left)	-	2	29 s	1 of each waveform	3 min	Open-label, crossover. *n* = 35	-	Stimulation was more effective in correcting postural imbalance in patients with right-hemispheric lesions than those with left-hemispheric lesions.
Babyar et al. (2018)	Bilateral mastoids	Non-zero mean sinusoidal (anodal-right or anodal-left)	1	2	15 min	1	-	Single-blind, crossover. *n* = 10	ramp up, ramp down and then 0 mA	Ipsilesional anodal GVS improved posturographic and inclinometry measures of pusher behavior
Tohyama et al. (2021)	Bilateral mastoids	DC (anodal-right or anodal-left)	-	1.5	30 s	22	1–3 days between each waveform	Single-blind, crossover. *n* = 24	0 mA	Influence of GVS on SVV and standing posture depended on the polarity and hemispheric lesion side
Tomioka et al. (2022)	Bilateral mastoids	DC (anodal-right or anodal-left)	-	1.5	Throughout duration of test (<20 min)	1 of each waveform	24 h	Single-blind, crossover. *n* = 9 for SSV experiment and *n* = 8 for COG experiment	ramp up, ramp down and then 0 mA	GVS improved SVV and COG

BIT, Behavioral Inattention Test; BLS, Burke Lateropulsion Scale; COG, Center of Gravity; PT, Physical Therapy; SCP, Scale for Contraversive Pushing; SPT, Smooth Pursuit Eye Movement training; STV, Subjective Tactile Vertical SVV, Subjective Visual Vertical; VST, Visual Scanning Training.

**Table 4:** Most common parameters and range of parameters used for post-stroke patients.

	Electrode montage	Waveform	Frequency (Hz)	Current intensity (mA)	Sham (Yes/No; procedure)	Length of stimulation session	Number of sessions	Inter-session/inter-test day interval
Most common parameter	Bilateral mastoids	DC (anodal-right or anodal-left)	Not applicable	1.5	Ramping up and down followed by 0 mA	20 min	No commonly used number of sessions	1 day
Range of parameters	Bilateral mastoids	DC (anodal-right or anodal-left), Noisy	Not applicable	0.3–1.5	No sham, 0 mA sham, or ramping up and down followed by 0 mA	29 s − 30 min	1–12	3 min–48 h

#### 3.2.1 Electrode montage, waveform, and frequency

Like GVS for improved balance, post-stroke studies have overwhelmingly preferred the bilateral-bipolar montage. However, while GVS studies aimed at improving postural control usually employ a noisy waveform, DC stimulation has been predominantly utilized for post-stroke patients, due to its ability to shift the subjective vertical (except for Wilkinson et al., 2014 who found improvement with a non-zero noisy waveform). Consequently, frequency is generally considered irrelevant in the context of GVS for post-stroke rehabilitation.

Regarding electrode polarity, earlier studies typically positioned the anodal electrode on the contralateral side of the lesion (Rorsman et al., 1999; Saj et al., 2006; Wilkinson et al., 2009). In contrast, more recent research has investigated the effects of placing the anodal electrode on either the ipsilesional or contralesional side for comparative purposes, usually a day apart to prevent carryover effects. As anticipated, these studies have often demonstrated greater symptom improvement when the anode was positioned contralaterally (Utz et al., 2011a; Nakamura et al., 2015; Babyar et al., 2018).

#### 3.2.2 Intensity and sham control

Similar to studies on improving postural control, GVS for post-stroke patients is often administered at an intensity just below the cutaneous sensory threshold to ensure that subjects remain naïve to the stimulation. In other studies, a set intensity was chosen, usually between 1 and 2 mA (Saj et al., 2006; Wilkinson et al., 2009; Utz et al., 2011a, etc.).

The application of DC results in perceptible sensation (starting ~0.3 mA) around the electrode sites for the first few minutes of stimulation. Subjects generally habituate to the sensation thereafter. Therefore, for supersensory studies, blinding was often achieved by delivering a brief stimulation current to the subjects in the sham arm and slowly ramping down the stimulation without the subject realizing. This 0 mA level is maintained for the rest of the session. Due to the habituation that occurs with prolonged DC exposure, the initial sensation, along with the subsequent ramp-down phase and eventual absence of sensation in the sham group, closely mirrored the experiences of subjects receiving active stimulation. However, consistent with studies on impaired postural control, the most common sham-blinding procedure in post-stroke GVS research involves placing electrodes on the mastoids, as in active stimulation, but withholding any current delivery.

#### 3.2.3 Stimulation sessions: length, number, and interval

Some studies tested the effect of stimulation on the performance of a specific task performed while stimulation was being delivered. In these studies, the stimulation length corresponded to the length of time it took the subject to perform the task (Rorsman et al., 1999; Saj et al., 2006; Tomioka et al., 2022; Schmidt et al., 2013a; Schmidt et al., 2013b). Other studies used the 20- or 30-min stimulation protocol (Zubko et al., 2013; Nakamura et al., 2014; Ruet et al., 2014) based on the safety protocol recommended for tDCS, as explained earlier.

Much like studies on postural control, GVS research involving post-stroke patients has largely concentrated on the effects of a single stimulation session, or in some cases, one session of each waveform spaced a day apart. Nevertheless, a subset of studies has explored the cumulative impact of repeated sessions, typically conducted with a one-day interval between each session. In both single and repeated session trials, most studies reported significant symptom improvements, with the exception of Krewer et al. (2013) and Ruet et al. (2014).

### 3.3 Other clinical applications

Motion sickness remains a frequent problem in the modern day as there are more frequent forms of passive motion (i.e., car, train, airplane) that occupy a substantial portion of individuals’ everyday life (Bertolini and Straumann, 2016). The onset of motion sickness includes feelings of uneasiness, and symptoms can rapidly progress to dizziness, nausea, vomiting, and loss of appetite. Motion sickness is a considerable health concern as some individuals may undergo a physiological habituation process, whereas others require lifelong motion sickness medications for their chronic susceptibility (Gutkovich et al., 2021). Currently, the most widely accepted theory on motion sickness is explained by the sensory conflict model which occurs when there are discrepancies between the proprioceptive, vestibular, and visual senses and the brain’s expectation of sensory signals. Gutkovich et al. tested the application of GVS to sailors susceptible to motion sickness (i.e., seasickness) Participants were restrained to a rotating chair and wore goggles that entirely restricted their vision. They received sinusoidal GVS in an inverse phase to chair rotation, anode stimulation applied toward the rotation side. The sailors receiving active GVS reported a decrease in seasickness symptoms upon their following sea voyages compared to the sham group (Gutkovich et al., 2021).

Simulated (virtual) environments also cause symptoms of motion sickness stemming from the mismatch between vestibular and visual inputs (“cybersickness”; Geyer and Biggs, 2018). This mismatch is primarily attributed to the same sensory conflict theory as motion sickness experienced while in passive motion (Reason, 1978; Oman, 1982; Oman, 1990). This mismatch induces motion sickness symptoms as well as compromising the immersive experience, and thereby the effectiveness of the virtual reality (VR) experience. Two different GVS methods have been investigated for the alleviation of cybersickness symptoms: matched-GVS and noisy GVS. For the first method, Cevette et al. applied matched-GVS using a novel electrode montage—two electrodes on each mastoid as well as one on the forehead and one at the back of the neck. Using this setup, they delivered stimulation over multiple independent “channels” to induce sensations of motion in multiple different directions corresponding to the direction and magnitude of the simulated motion in VR. The direction of GVS-induced perceived rotation depended on the direction of current between each of the four electrodes. By matching vestibular cues to visual cues, the gap of visual-vestibular sensory incongruence is resolved, intending to alleviate cybersickness symptoms (Cevette et al., 2012). The second method (noisy GVS) sends zero-mean white noise to potentially facilitate sensory re-weighting. A possible explanation provided by the authors is that a noisy vestibular stimulus rapidly reduces vestibular cue reliability, causing visual self-motion cues to be up-weighted in return (Weech et al., 2020). In a similar fashion, Dilda et al. delivered a sum-of-sines (i.e., multisine) waveform to healthy volunteers and found that it trained them to up-weight non-vestibular cues, suggesting that GVS could serve as an effective pre-flight training method for preparing astronauts to manage vestibular perturbations experienced during spaceflight (Dilda et al., 2014).

Recently, GVS has been explored as a method to promote sleep (Kishi et al., 2023; Goothy and McKeown, 2021). Kishi et al. (2023) induced a rocking sensation through sinusoidal GVS current to facilitate sleep, a phenomenon similar to the findings of Bayer et al. (2011) who demonstrated that rocking helps synchronize brain waves, reinforcing endogenous sleep rhythms—much like the traditional practice of rocking infants to sleep. In 2023, the U.S. Food and Drug Administration (FDA) approved the first GVS-based device for therapeutic use: the Modius Sleep device, for treating chronic insomnia (Food and Drug Administration, 2023). However, the Modius Sleep device uses symmetrical biphasic rectangular waveforms, similar to those used in cranial electrotherapy stimulation (CES) devices, rather than the sinusoidal GVS current typically employed to induce a rocking sensation. CES devices traditionally deliver stimulation through electrodes placed on the temples, earlobes, or in the ear and are commonly used to treat conditions such as anxiety and depression (Datta et al., 2013; Brunyé et al., 2021). Given that biphasic rectangular pulses are not traditionally used in GVS, it is possible that it was selected for the Modius Sleep device to leverage CES as a predicate for securing 510(k) clearance by aligning with the established CES regulatory pathway, rather than due to its ideal properties for GVS. The stimulation parameters used for motion sickness studies as well as other treatment indications are enumerated in Table 5.

**Table 5:** GVS stimulation parameters investigated for other clinical applications and their clinical outcomes.

Study	Electrode montage	Waveform	Frequency (Hz)	Current intensity (mA; mean ± SD)	Length of stimulation session	Number of sessions	Inter-session/Inter-test day interval	Study design and size	Sham procedure	Clinical outcome
Cervical dystonia										
Rosengren and Colebatch (2006)	Bilateral mastoids	20 msec DC pulses (anodal-right or anodal-left)	3	1.5 or 2.5	50 s	4	2 min	Open-label. *n* = 1	-	GVS reduced neck muscle activity
Cybersickness or motion sickness										
Cevette et al. (2012)	Mastoids, forehead, and back of neck (Multi-channel)	DC	-	≤ 2.5	When experiencing visual stimulus	1	-	Single-blind, crossover. *n* = 21	1 mA constant current	Significant decrease in nausea, dizziness, and peripheral symptoms
Weech et al. (2020)	Bilateral mastoids	Noisy	≤ 100	1.75	30 min	1	-	Single-blind, crossover. *n* = 44	0 mA	Significant decrease in FMS score in the intense VR group, but not in SSQ scores or in the moderate VR group
Gutkovich et al. (2021)	Bilateral mastoids	Sine	0.017	75% of discomfort level	20 min	4	2 sessions per day separated by a 5 min break	Single blind. Active *n* = 15, sham *n* = 15	0 mA	Significant decrease in vestibular time constant and seasickness over 3 months following stimulation
Experimental Pain										
Hagiwara et al., 2020	Bilateral mastoids	DC (anodal-right or anodal-left)	-	2	7 min	1	-	Single-blind, crossover. *n* = 16	ramp up, ramp down, and then 0 mA	Significant decrease in Verbal Numerical Ratings of pain. Right-anodal decreased laser-evoked potentials, but left-anodal did not.
Type II Diabetes Mellitus										
Lotfi et al. (2021b)	Bilateral mastoids	Noisy	1–30	Maximum subthreshold	20 min	36	Three times a week	Single-blind. Active *n* = 16; sham *n* = 16; control *n* = 16	Short stimulation interval followed by ramp down and 0 mA	Statistically significant reduction in blood sugar level and BMI

VAS-A, visual analog scales measuring state anxiety; SSQ, simulator sickness questionnaire; FMS, fast motion sickness scale.

## 4 Conclusion

The studies discussed demonstrate that there is a notable divergence in the selection of stimulation parameters. Some parameters are widely accepted, while others remain poorly defined and require further investigation to identify the most effective settings. Studies on impaired balance usually employed noisy waveforms, driven by the beneficial effects of input noise in enhancing neural system sensitivity. This occurs through noise-enhanced responses of nonlinear systems to weak signals, a phenomenon known as stochastic resonance (Wiesenfeld and Moss, 1995). It is hypothesized that a central circuit involved in signaling movement initiation, with a pathologically elevated threshold, may benefit from noisy modulation of afferent firing rates (Pan et al., 2008). Conversely, for treating impaired verticality, studies often employed DC stimulation to counteract the misalignment (Oppenländer et al., 2015a).

With respect to current intensity, the choice has varied between choosing an individualized optimal intensity (i.e., maximizing an individual’s performance on a task), individualized subthreshold intensity, or a fixed intensity. This decision is guided by the exact outcome measure being tested and as applicable, highlights the need for personalization, as the same intensity can elicit different vestibular sensations and physiological effects in different subjects, including within the same patient population.

As expected, the choice of waveform has a bearing on the length of the stimulation session. As noisy GVS at imperceptible levels does not cause noticeable side effects in the form of oculomotor and postural responses, some studies have delivered stimulation spanning hours to an entire day. When using DC, studies have generally not exceeded the 30 min duration and inter-session duration of 1 day based on the safety criteria and protocols in place for the more thoroughly researched tDCS modality.

Repeated applications leading to cumulative and/or sustaining effects are well documented in other brain stimulation modalities (e.g., Razza et al., 2020; Black et al., 2023; Valter et al., 2024). However, most GVS studies have been limited to single session protocols. As such, there is a gap in our understanding of the long-term and cumulative effects of GVS. This highlights the need for comprehensive research to establish the appropriate treatment indication and protocol, and to examine the cumulative and long-term impacts of GVS.

In conclusion, certain GVS parameters are well-accepted for specific applications, such as the need to deliver a noisy stimulus waveform to improve postural control in Bilateral Vestibulopathy, whereas other parameters still lack consensus, such as the number of stimulation sessions necessary for improving spatial neglect in post-stroke patients. Also, a wide variety in noise frequency ranges have been used for improving postural control in subjects with vestibular disorders. While the wide range makes it difficult to determine the most effective frequency band for a given indication, it also demonstrates that a wide range of parameters are biologically active. Additional studies are therefore necessary to clarify the optimal frequency for each population and application.

GVS continues to hold significant potential for clinical utility. Furthermore, owing to low intensity current needs, GVS devices are portable and can be therefore administered across a wide variety of settings such as laboratory, clinical and home locations, mimicking protocols developed in other non-invasive brain stimulation modalities (Valter et al., 2021; Pilloni et al., 2022; Black et al., 2023). Research has also demonstrated that GVS application is even feasible in spaceflight due to its low mass and power requirements, low power usage, and minimal side effects (Putman et al., 2021; Sherman S. et al., 2023; Sherman S. O. et al., 2023). Given the safety profile, protocols can explore delivering multiple sessions over weeks and thereby expect developing clinical utility in myriad conditions. Our review serves as a suggestion for future GVS research protocols and clinical applications with respect to the choice of stimulation parameters and related considerations for specific patient populations and conditions.

Results of this review indicate that the field of GVS is still evolving, with no universally standard application guidelines. Instead, current evidence appears to be condition-dependent or patient population-specific, limiting making conclusions on an overall all-cause acceptable GVS parameter protocol, but does align more favorably with the changing clinical landscape of precision medicine practices. Nevertheless, elucidative information has surfaced from the current review that serves as the basis for early key recommendations, critical to moving the field of GVS forward. The following are those recommendations that scientists can use as reference when designing GVS research studies. First, it is essential to perform (at least one) sham condition, appropriate as a control condition for the study’s scientific objectives, and provide sufficient methodological details regarding the sham (e.g., was current applied away from the mastoids, was no current applied, were electrodes on, was the subject blinded to the GVS application, etc.). This is particularly critical for studies investigating after-effects or the longitudinal application of GVS, where there is otherwise a confound between the presence of GVS and time (which could impact learning, fatigue, boredom, etc.). We also encourage investigators, particularly for subthreshold GVS, to assess whether subjects are able to feel tingling sensations or other side effects from the GVS application, in a two-alternative forced choice test (where GVS is presented in one interval and not in the other, and the subject is required to guess which interval contained the GVS), and investigators ensure the recognition performance is no better than chance.

Second, while the literature suggests that GVS applications may require (or benefit) from personalization to each individual, this can incidentally provide investigators excessive opportunities for identifying a positive effect of GVS. For example, testing each subject with multiple amplitudes of GVS (to identify which is preferred for each individual) and then comparing each individual’s optimal amplitude to a sham condition can increase potential for false positive findings. Personalization procedures therefore need to be carefully pre-defined to ensure scientific rigor. Third, future research should aim to systematically compare multiple GVS applications (waveform, frequency, intensity, etc) in the same study for a given patient population and outcome. Nearly all prior research has only assessed one particular GVS application, requiring the field to compare across studies, performed in different laboratories, with nuanced differences in assessments, metrics, timing, and other procedures. Current, variegated approaches across studies preclude determining ‘best’ sets of GVS parameters given a specific use case. Finally, to aid in standardized GVS applications across studies, we encourage the publication of null result outcomes from GVS studies. By publishing these findings, more comparisons can be made between GVS applications that otherwise may not be noted in the literature. Further, this approach will provide advanced guidance on what GVS applications may not be useful for specific patient populations and outcomes.

## References

[ref1] Ankit J. Aparna S. Meena G. (2020). Effect of vestibular stimulation on postural stability and mobility in elderly. Indian J. For. Med. Toxicol. 14, 9269–9278. doi: 10.37506/ijfmt.v14i4.13197

[ref2] Aoyama K. Higuchi D. Sakurai K. Maeda T. Ando H. (2017). “GVS RIDE: providing a novel experience using a head mounted display and four-pole galvanic vestibular stimulation” in ACM SIGGRAPH 2017 emerging technologies (New York, NY, United States: Association for Computing Machinery), 1–2.

[ref3] Assländer L. Giboin L. S. Gruber M. Schniepp R. Wuehr M. (2021). No evidence for stochastic resonance effects on standing balance when applying noisy galvanic vestibular stimulation in young healthy adults. Sci. Rep. 11:12327. doi: 10.1038/s41598-021-91808-w, PMID: 34112904 PMC8192540

[ref4] Babyar S. Santos T. Will-Lemos T. Mazin S. Edwards D. Reding M. (2018). Sinusoidal transcranial direct current versus galvanic vestibular stimulation for treatment of lateropulsion poststroke. J. Stroke Cerebrovasc. Dis. 27, 3621–3625. doi: 10.1016/j.jstrokecerebrovasdis.2018.08.034, PMID: 30314762

[ref5] Barra J. Marquer A. Joassin R. Reymond C. Metge L. Chauvineau V.. (2010). Humans use internal models to construct and update a sense of verticality. Brain J. Neurol. 133, 3552–3563. doi: 10.1093/brain/awq311, PMID: 21097492

[ref6] Bayer L. Constantinescu I. Perrig S. Vienne J. Vidal P. P. Mühlethaler M.. (2011). Rocking synchronizes brain waves during a short nap. Curr. Biol. 21, R461–R462. doi: 10.1016/j.cub.2011.05.012, PMID: 21683897

[ref7] Bertolini G. Straumann D. (2016). Moving in a moving world: a review on vestibular motion sickness. Front. Neurol. 7:14. doi: 10.3389/fneur.2016.00014, PMID: 26913019 PMC4753518

[ref8] Bikson M. Grossman P. Thomas C. Zannou A. L. Jiang J. Adnan T.. (2016). Safety of transcranial direct current stimulation: evidence based update 2016. Brain Stimul. 9, 641–661. doi: 10.1016/j.brs.2016.06.004, PMID: 27372845 PMC5007190

[ref9] Bindman L. J. Lippold O. C. J. Redfearn J. (1964). The action of brief polarizing currents on the cerebral cortex of the rat (1) during current flow and (2) in the production of long-lasting after-effects. J. Physiol. 172, 369–382. doi: 10.1113/jphysiol.1964.sp007425, PMID: 14199369 PMC1368854

[ref10] Black B. Hunter S. Cottrell H. Dar R. Takahashi N. Ferguson B. J.. (2023). Remotely supervised at-home delivery of ta VNS for autism spectrum disorder: feasibility and initial efficacy. Front. Psychol. 14:1238328. doi: 10.3389/fpsyt.2023.1238328, PMID: 37840787 PMC10568329

[ref11] Bonan I. Leblong E. Leplaideur S. Laviolle B. Ponche S. T. Yelnik A. P. (2016). The effect of optokinetic and galvanic vestibular stimulations in reducing post-stroke postural asymmetry. Clin. Neurophysiol. 127, 842–847. doi: 10.1016/j.clinph.2015.03.026, PMID: 26051751

[ref12] Breuer J. (1874). Ueber die Funktion der Bogengänge des Ohrlabyrinths. Med. Jahrb 4, 72–124.

[ref13] Brunyé T. T. Patterson J. E. Wooten T. Hussey E. K. (2021). A critical review of cranial electrotherapy stimulation for neuromodulation in clinical and non-clinical samples. Front. Hum. Neurosci. 15:625321. doi: 10.3389/fnhum.2021.625321, PMID: 33597854 PMC7882621

[ref14] Brunyé T. T. Navarro E. Hart-Pomerantz H. Valter Y. Datta A. Taylor H. A. (2024). Guiding human navigation with noninvasive vestibular stimulation and evoked Mediolateral sway. J. Cogn. Enhanc. 8, 54–64. doi: 10.1007/s41465-023-00283-w, PMID: 39808227

[ref15] Cai J. Lee S. Ba F. Garg S. Kim L. J. Liu A.. (2018). Galvanic vestibular stimulation (GVS) augments deficient Pedunculopontine nucleus (PPN) connectivity in mild Parkinson's disease: fMRI effects of different stimuli. Front. Neurosci. 12:101. doi: 10.3389/fnins.2018.00101, PMID: 29541016 PMC5835530

[ref16] Carmona S. Ferrero A. Pianetti G. Escolá N. Arteaga M. V. Frankel L. (2011). Galvanic vestibular stimulation improves the results of vestibular rehabilitation. Ann. N. Y. Acad. Sci. 1233, E1–E7. doi: 10.1111/j.1749-6632.2011.06269.x, PMID: 22360772

[ref17] Cevette M. J. Stepanek J. Cocco D. Galea A. M. Pradhan G. N. Wagner L. S.. (2012). Oculo-vestibular recoupling using galvanic vestibular stimulation to mitigate simulator sickness. Aviat. Space Environ. Med. 83, 549–555. doi: 10.3357/ASEM.3239.2012, PMID: 22764608

[ref18] Ceylan D. Ş. Ataş A. Kaya M. (2020). The effect of galvanic vestibular stimulation in the rehabilitation of patients with vestibular disorders. ORL 83, 25–34. doi: 10.1159/000509971, PMID: 32992316

[ref19] Chapman EK Gagliuso AH Rice DL Rajguru SM Martinelli GP Holstein GR. (2019). Comparison of vestibular nucleus neurons activated by unilateral electrical stimulation of the posterior canal nerve and by pulsed infrared laser stimulation (pIR) of the posterior canal (abstract). Association for Research in otolaryngology 42nd annual mid winter meeting. Baltimore, MD. p. 52.

[ref21] Datta A. Dmochowski J. P. Guleyupoglu B. Bikson M. Fregni F. (2013). Cranial electrotherapy stimulation and transcranial pulsed current stimulation: a computer based high-resolution modeling study. NeuroImage 65, 280–287. doi: 10.1016/j.neuroimage.2012.09.062, PMID: 23041337

[ref22] Dilda V. Morris T. R. Yungher D. A. Mac Dougall H. G. Moore S. T. (2014). Central adaptation to repeated galvanic vestibular stimulation: implications for pre-flight astronaut training. PLoS One 9:e112131. doi: 10.1371/journal.pone.0112131, PMID: 25409443 PMC4237321

[ref23] Dlugaiczyk J. Gensberger K. D. Straka H. (2019). Galvanic vestibular stimulation: from basic concepts to clinical applications. J. Neurophysiol. 121, 2237–2255. doi: 10.1152/jn.00035.2019, PMID: 30995162

[ref24] Eder J. Kellerer S. Amberger T. Keywan A. Dlugaiczyk J. Wuehr M.. (2022). Combining vestibular rehabilitation with noisy galvanic vestibular stimulation for treatment of bilateral vestibulopathy. J. Neurol. 269, 5731–5737. doi: 10.1007/s00415-022-11033-x, PMID: 35212789 PMC9553809

[ref25] Fitzpatrick R. C. Day B. L. (2004). Probing the human vestibular system with galvanic stimulation. J. Appl. Physiol. 96, 2301–2316. doi: 10.1152/japplphysiol.00008.2004, PMID: 15133017

[ref26] Food and Drug Administration (2023). Center for Devices and Radiological Health, K230826 summary letter, Available at: https://www.accessdata.fda.gov/cdrh_docs/pdf23/K230826.pdf (Accessed March 28, 2024)

[ref27] Forbes P. A. Kwan A. Mitchell D. E. Blouin J. S. Cullen K. E. (2023). The neural basis for biased behavioral responses evoked by galvanic vestibular stimulation in primates. J. Neurosci. 43, 1905–1919. doi: 10.1523/JNEUROSCI.0987-22.2023, PMID: 36732070 PMC10027042

[ref28] Fujimoto C. Yamamoto Y. Kamogashira T. Kinoshita M. Egami N. Uemura Y.. (2016). Noisy galvanic vestibular stimulation induces a sustained improvement in body balance in elderly adults. Sci. Rep. 6:37575. doi: 10.1038/srep37575, PMID: 27869225 PMC5116631

[ref29] Fujimoto C. Egami N. Kawahara T. Uemura Y. Yamamoto Y. Yamasoba T.. (2018). Noisy galvanic vestibular stimulation sustainably improves posture in bilateral Vestibulopathy. Front. Neurol. 9:900. doi: 10.3389/fneur.2018.00900, PMID: 30405522 PMC6204397

[ref30] Fujimoto C. Kawahara T. Kinoshita M. Ichijo K. Oka M. Kamogashira T.. (2021). Minimally important differences for subjective improvement in postural stability in patients with bilateral vestibulopathy. Neurosci. Lett. 747:135706. doi: 10.1016/j.neulet.2021.135706, PMID: 33548406

[ref31] Galvani L. (1953). De viribus electricitatis in motu musculari commentarius, Bologna, Italy: Instituti Scientiarum, 1791. Translated by Foley MG as Commentary on the effects of electricity on muscular motion. Norwalk, CT: Burndy Library.

[ref32] Galvan-Garza R. C. Clark T. K. Mulavara A. P. Oman C. M. (2018). Exhibition of stochastic resonance in vestibular tilt motion perception. Brain Stimul. 11, 716–722. doi: 10.1016/j.brs.2018.03.017, PMID: 29656906

[ref33] Gaspar A. G. M. Lapão L. V. (2021). eHealth for addressing balance disorders in the elderly: systematic review. J. Med. Internet Res. 23:e22215. doi: 10.2196/22215, PMID: 33908890 PMC8116987

[ref34] Gensberger K. D. Kaufmann A. K. Dietrich H. Branoner F. Banchi R. Chagnaud B. P.. (2016). Galvanic vestibular stimulation: cellular substrates and response patterns of neurons in the vestibulo-ocular network. J. Neurosci. 36, 9097–9110. doi: 10.1523/JNEUROSCI.4239-15.2016, PMID: 27581452 PMC6601907

[ref35] Geyer D. J. Biggs A. T. (2018). The persistent issue of simulator sickness in naval aviation training. Aerosp. Med. Hum. Perform. 89, 396–405. doi: 10.3357/AMHP.4906.2018, PMID: 29562971

[ref36] Goel R. Kofman I. Jeevarajan J. De Dios Y. Cohen H. S. Bloomberg J. J.. (2015). Using low levels of stochastic vestibular stimulation to improve balance function. PLoS One 10:E0136335. doi: 10.1371/Journal.Pone.0136335, PMID: 26295807 PMC4546608

[ref37] Goothy S. S. K. McKeown J. (2021). Modulation of sleep using electrical vestibular nerve stimulation prior to sleep onset: a pilot study. J. Basic Clin. Physiol. Pharmacol. 32, 19–23. doi: 10.1515/jbcpp-2020-0019, PMID: 33006952

[ref38] Gutkovich Y. E. Lagami D. Jamison A. Fonar Y. Tal D. (2021). Galvanic vestibular stimulation as a novel treatment for seasickness. Exp. Brain Res. 240, 429–437. doi: 10.1007/s00221-021-06263-w, PMID: 34782915

[ref39] Hagiwara K. Perchet C. Frot M. Bastuji H. Garcia-Larrea L. (2020). Cortical modulation of nociception by galvanic vestibular stimulation: a potential clinical tool? Brain Stimul. 13, 60–68. doi: 10.1016/j.brs.2019.10.009, PMID: 31636023

[ref40] Heilman K. M. Valenstein E. Watson R. T. (2000). Neglect and related disorders. Semin. Neurol. 20, 463–470. doi: 10.1055/s-2000-13179, PMID: 11149702

[ref41] Hirasaki E. Moore S. T. Raphan T. Cohen B. (1999). Effects of walking velocity on vertical head and body movements during locomotion. Exp. Brain Res. 127, 117–130. doi: 10.1007/s002210050781, PMID: 10442403

[ref42] Hodgson P. Stephensen D. Wilkinson D. (2021). Galvanic vestibular stimulation and balance control in Parkinson’s disease. Physiotherapy 113:e136. doi: 10.1016/j.physio.2021.10.127, PMID: 39812234

[ref43] Holstein G. R. Friedrich V. L. Jr. Martinelli G. P. Ogorodnikov D. Yakushin S. B. Cohen B. (2012). Fos expression in neurons of the rat vestibulo-autonomic pathway activated by sinusoidal galvanic vestibular stimulation. Front. Neurol. 3:4. doi: 10.3389/fneur.2012.00004, PMID: 22403566 PMC3289126

[ref44] Inukai Y. Masaki M. Otsuru N. Saito K. Miyaguchi S. Kojima S.. (2018). Effect of noisy galvanic vestibular stimulation in community-dwelling elderly people: a randomised controlled trial. J. Neuroeng. Rehabil. 15:63. doi: 10.1186/s12984-018-0407-6, PMID: 29970144 PMC6029379

[ref45] Iwasaki S. Yamamoto Y. Togo F. Kinoshita M. Yoshifuji Y. Fujimoto C.. (2014). Noisy vestibular stimulation improves body balance in bilateral vestibulopathy. Neurology 82, 969–975. doi: 10.1212/WNL.0000000000000215, PMID: 24532279

[ref46] Iwasaki S. Fujimoto C. Egami N. Kinoshita M. Togo F. Yamamoto Y.. (2018). Noisy vestibular stimulation increases gait speed in normals and in bilateral vestibulopathy. Brain Stimul. 11, 709–715. doi: 10.1016/j.brs.2018.03.005, PMID: 29563049

[ref47] Karnath H. O. (1994). Disturbed coordinate transformation in the neural representation of space as the crucial mechanism leading to neglect. Neuropsychol. Rehabil. 4, 147–150. doi: 10.1080/09602019408402273

[ref48] Kazemi A. Mirian M. S. Lee S. McKeown M. J. (2021). Galvanic vestibular stimulation effects on EEG biomarkers of motor vigor in Parkinson's disease. Front. Neurol. 12:759149. doi: 10.3389/fneur.2021.759149, PMID: 34803892 PMC8599939

[ref49] Kerkhoff G. Hildebrandt H. Reinhart S. Kardinal M. Dimova V. Utz K. S. (2011). A long-lasting improvement of tactile extinction after galvanic vestibular stimulation: two sham-stimulation controlled case studies. Neuropsychologia 49, 186–195. doi: 10.1016/j.neuropsychologia.2010.11.014, PMID: 21094654

[ref50] Keywan A. Wuehr M. Pradhan C. Jahn K. (2018). Noisy galvanic stimulation improves roll-tilt vestibular perception in healthy subjects. Front. Neurol. 9:83. doi: 10.3389/fneur.2018.00083, PMID: 29545766 PMC5837962

[ref51] Khoshnam M. Häner D. M. C. Kuatsjah E. Zhang X. Menon C. (2018). Effects of galvanic vestibular stimulation on upper and lower extremities motor symptoms in Parkinson’s disease. Front. Neurosci. 12:633. doi: 10.3389/fnins.2018.00633, PMID: 30254564 PMC6141687

[ref52] Kim K. S. Minor L. B. Santina C. C. D. Lasker D. M. (2011). Variation in response dynamics of regular and irregular vestibular-nerve afferents during sinusoidal head rotations and currents in the chinchilla. Exp. Brain Res. 210, 643–649. doi: 10.1007/s00221-011-2600-8, PMID: 21369854 PMC4010622

[ref53] Kishi A. Togo F. Yamamoto Y. (2023). Slow-oscillatory galvanic vestibular stimulation promotes sleep in healthy young adults. Brain Stimul. 16, 298–299. doi: 10.1016/j.brs.2023.01.535

[ref54] Ko L-W Chikara RK Chen P-Y Jheng Y-C Wang C-C Yang Y-C. (2020). Noisy galvanic vestibular stimulation (stochastic resonance) changes electroencephalography activities and postural control in patients with bilateral vestibular. 10.3390/brainsci10100740 PMC7602631 33076417

[ref55] Krewer C. Rieß K. Bergmann J. Müller F. Jahn K. Koenig E. (2013). Immediate effectiveness of single-session therapeutic interventions in pusher behaviour. Gait Posture 37, 246–250. doi: 10.1016/j.gaitpost.2012.07.014, PMID: 22889929

[ref56] Kwan A. Forbes P. A. Mitchell D. E. Blouin J. S. Cullen K. E. (2019). Neural substrates, dynamics and thresholds of galvanic vestibular stimulation in the behaving primate. Nat. Commun. 10:1904. doi: 10.1038/s41467-019-09738-1, PMID: 31015434 PMC6478681

[ref57] Lee S. Kim D. J. Svenkeson D. Parras G. Oishi M. M. McKeown M. J. (2015). Multifaceted effects of noisy galvanic vestibular stimulation on manual tracking behavior in Parkinson’s disease. Front. Syst. Neurosci. 9:5. doi: 10.3389/fnsys.2015.00005, PMID: 25698944 PMC4313776

[ref58] Lee S. Smith P. F. Lee W. H. McKeown M. J. (2021a). Frequency-specific effects of galvanic vestibular stimulation on response-time performance in Parkinson’s disease. Front. Neurol. 12:758122. doi: 10.3389/fneur.2021.758122, PMID: 34795633 PMC8593161

[ref59] Lee S. Liu A. McKeown M. J. (2021b). Current perspectives on galvanic vestibular stimulation in the treatment of Parkinson's disease. Expert. Rev. Neurother. 21, 405–418. doi: 10.1080/14737175.2021.1894928, PMID: 33621149

[ref60] Liu A. Bi H. Li Y. Lee S. Cai J. Mi T.. (2021). Galvanic vestibular stimulation improves subnetwork interactions in Parkinson’s disease. J. Healthc. Eng. 2021, 1–11. doi: 10.1155/2021/6632394, PMID: 34094040 PMC8137296

[ref61] Lobel E. Kleine J. F. Leroy-Willig A. Van de Moortele P. F. Le Bihan D. Grüsser O. J.. (1999). Cortical areas activated by bilateral galvanic vestibular stimulation. Ann. N. Y. Acad. Sci. 871, 313–323. doi: 10.1111/j.1749-6632.1999.tb09194.x, PMID: 10372081

[ref62] Lajoie K. Marigold D. S. Valdés B. A. Menon C. (2021). The potential of noisy galvanic vestibular stimulation for optimizing and assisting human performance. Neuropsychologia 152:107751. doi: 10.1016/j.neuropsychologia.2021.107751, PMID: 33434573

[ref63] Lotfi Y. Farahani A. Azimiyan M. Moossavi A. Bakhshi E. (2021a). Comparison of efficacy of vestibular rehabilitation and noisy galvanic vestibular stimulation to improve dizziness and balance in patients with multiple sclerosis. J. Vestib. Res. 31, 541–551. doi: 10.3233/VES-201609, PMID: 33967075

[ref64] Lotfi Y. Absalan A. Keykhaei M. A. (2021b). Investigation of effects of galvanic vestibular stimulation on patients with type 2 diabetes. Crescent J. Med. Biol. Sci. 8, 174–178.

[ref65] McLaren R. Smith P. F. Taylor R. L. Niazi I. K. Taylor D. (2023). Scoping out noisy galvanic vestibular stimulation: a review of the parameters used to improve postural control. Front. Neurosci. 17:1156796. doi: 10.3389/fnins.2023.1156796, PMID: 37205050 PMC10187481

[ref66] Mulavara A. P. Fiedler M. J. Kofman I. S. Wood S. J. Serrador J. M. Peters B.. (2011). Improving balance function using vestibular stochastic resonance: optimizing stimulus characteristics. Exp. Brain Res. 210, 303–312. doi: 10.1007/s00221-011-2633-z, PMID: 21442221

[ref67] Mulavara A. P. Kofman I. S. De Dios Y. E. Miller C. Peters B. T. Goel R.. (2015). Using low levels of stochastic vestibular stimulation to improve locomotor stability. Front. Syst. Neurosci. 9:117. doi: 10.3389/fnsys.2015.00117, PMID: 26347619 PMC4547107

[ref68] Nakamura J. Kita Y. Yuda T. Ikuno K. Okada Y. Shomoto K. (2014). Effects of galvanic vestibular stimulation combined with physical therapy on pusher behavior in stroke patients: a case series. Neuro Rehabil. 35, 31–37. doi: 10.3233/NRE-141094, PMID: 24990006

[ref69] Nakamura J. Kita Y. Ikuno K. Kojima K. Okada Y. Shomoto K. (2015). Influence of the stimulus parameters of galvanic vestibular stimulation on unilateral spatial neglect. Neuroreport 26, 462–466. doi: 10.1097/WNR.0000000000000369, PMID: 25875473

[ref70] Nguyen T. T. Nam G. S. Kang J. J. Han G. C. Kim J. S. Dieterich M.. (2021). Galvanic vestibular stimulation improves spatial cognition after unilateral Labyrinthectomy in mice. Front. Neurol. 12:716795. doi: 10.3389/fneur.2021.716795, PMID: 34393985 PMC8358680

[ref71] Nguyen T. T. Lee S. B. Kang J. J. Oh S. Y. (2023). Optimal design of galvanic vestibular stimulation for patients with vestibulopathy and cerebellar disorders. Brain Sci. 13:1333. doi: 10.3390/brainsci13091333, PMID: 37759934 PMC10526825

[ref72] Nooristani M. Bigras C. Lafontaine L. Bacon B.-A. Maheu M. Champoux F. (2021). Vestibular function modulates the impact of nGVS on postural control in older adults. J. Neurophysiol. 125, 489–495. doi: 10.1152/jn.00512.2020, PMID: 33296620

[ref73] Okada Y. Kita Y. Nakamura J. Kataoka H. Kiriyama T. Ueno S.. (2015). Galvanic vestibular stimulation may improve anterior bending posture in Parkinson’s disease. Neuroreport 26, 405–410. doi: 10.1097/WNR.0000000000000360, PMID: 25793635

[ref74] Oman C. M. (1982). A heuristic mathematical model for the dynamics of sensory conflict and motion sickness. Acta Otolaryngol. Suppl. 392, 1–44. doi: 10.3109/00016488209108197, PMID: 6303041

[ref75] Oman C. M. (1990). Motion sickness: a synthesis and evaluation of the sensory conflict theory. Can. J. Physiol. Pharmacol. 68, 294–303. doi: 10.1139/y90-044, PMID: 2178753

[ref76] Oppenländer K. Utz K. S. Reinhart S. Keller I. Kerkhoff G. Schaadt A. K. (2015a). Subliminal galvanic-vestibular stimulation recalibrates the distorted visual and tactile subjective vertical in right-sided stroke. Neuropsychologia 74, 178–183. doi: 10.1016/j.neuropsychologia.2015.03.004, PMID: 25744870

[ref77] Oppenländer K. Keller I. Karbach J. Schindler I. Kerkhoff G. Reinhart S. (2015b). Subliminal galvanic-vestibular stimulation influences ego-and object-centred components of visual neglect. Neuropsychologia 74, 170–177. doi: 10.1016/j.neuropsychologia.2014.10.039, PMID: 25445776

[ref78] Page M. J. McKenzie J. E. Bossuyt P. M. Boutron I. Hoffmann T. C. Mulrow C. D.. (2021). The PRISMA 2020 statement: an updated guideline for reporting systematic reviews. BMJ 372:n71. doi: 10.1136/bmj.n71, PMID: 33782057 PMC8005924

[ref79] Pan W. Soma R. Kwak S. Yamamoto Y. (2008). Improvement of motor functions by noisy vestibular stimulation in central neurodegenerative disorders. J. Neurol. 255, 1657–1661. doi: 10.1007/s00415-008-0950-3, PMID: 18677633

[ref80] Pérez-Fernández N. Ramos-Macías A. (2023). Recent advances in the diagnosis and treatment of vestibular disorders. J. Clin. Med. 12:5281. doi: 10.3390/jcm12165281, PMID: 37629323 PMC10455078

[ref81] Pilloni G. Vogel-Eyny A. Lustberg M. Best P. Malik M. Walton-Masters L.. (2022). Tolerability and feasibility of at-home remotely supervised transcranial direct current stimulation (RS-tDCS): single-center evidence from 6, 779 sessions. Brain Stimul. 15, 707–716. doi: 10.1016/j.brs.2022.04.014, PMID: 35470019

[ref82] Purkinje J. (1820). Beiträge zur näheren Kenntnis des Schwindels. Med. Jahrb d k k österr Staates Wien 6, 23–35.

[ref83] Putman E. Galvan-Garza R. C. Clark T. K. (2021). The effect of Noisy galvanic vestibular stimulation on learning of functional mobility and manual control nulling sensorimotor tasks. Front. Hum. Neurosci. 15:756674. doi: 10.3389/fnhum.2021.756674, PMID: 34803637 PMC8595260

[ref84] Razza L. B. Palumbo P. Moffa A. H. Carvalho A. F. Solmi M. Loo C. K.. (2020). A systematic review and meta-analysis on the effects of transcranial direct current stimulation in depressive episodes. Depress. Anxiety 37, 594–608. doi: 10.1002/da.23004, PMID: 32101631

[ref85] Reason J. (1978). Motion sickness: some theoretical and practical considerations. Appl. Ergon. 9, 163–167. doi: 10.1016/0003-6870(78)90008-X, PMID: 15677267

[ref86] Rorsman I. Magnusson M. Johansson B. B. (1999). Reduction of visuo-spatial neglect with vestibular galvanic stimulation. Scand. J. Rehabil. Med. 31, 117–124. doi: 10.1080/003655099444632, PMID: 10380728

[ref87] Rosengren S. M. Colebatch J. G. (2006). Cervical dystonia responsive to acoustic and galvanic vestibular stimulation. Movement Disord. 21, 1495–1499. doi: 10.1002/mds.20982, PMID: 16758481

[ref88] Ruet A. Jokic C. Denise P. Leroy F. Azouvi P. (2014). Does galvanic vestibular stimulation reduce spatial neglect? A negative study. Ann. Phys. Rehabil. Med. 57, 570–577. doi: 10.1016/j.rehab.2014.09.009, PMID: 25447749

[ref89] Sabzevar F. T. Vautrelle N. Zheng Y. Smith P. F. (2023). Vestibular modulation of the tail of the rat striatum. Sci. Rep. 13:4443. doi: 10.1038/s41598-023-31289-1, PMID: 36932124 PMC10023713

[ref90] Saeys W. Herssens N. Verwulgen S. Truijen S. (2018). Sensory information and the perception of verticality in post-stroke patients. Another point of view in sensory reweighting strategies. PLoS One 13:e0199098. doi: 10.1371/journal.pone.0199098, PMID: 29958286 PMC6025873

[ref91] Saj A. Honoré J. Rousseaux M. (2006). Perception of the vertical in patients with right hemispheric lesion: effect of galvanic vestibular stimulation. Neuropsychologia 44, 1509–1512. doi: 10.1016/j.neuropsychologia.2005.11.018, PMID: 16414094

[ref92] Sherman S. Jonsen A. Lewis Q. Schlittenhart M. Szafir D. Clark T. K.. (2023). Training augmentation using additive sensory noise in a lunar rover navigation task. Front. Neurosci. 17:1180314. doi: 10.3389/fnins.2023.1180314, PMID: 37424995 PMC10326282

[ref93] Sherman S. O. Shen Y. Gutierrez-Mendoza D. Schlittenhart M. Watson C. Clark T. K.. (2023). Additive sensory noise effects on operational performance in a landing simulation. Aerosp. Med. Hum. Perform. 94, 770–779. doi: 10.3357/AMHP.6251.2023, PMID: 37726913

[ref94] Schmidt L. Utz K. S. Depper L. Adams M. Schaadt A. K. Reinhart S.. (2013a). Now you feel both: galvanic vestibular stimulation induces lasting improvements in the rehabilitation of chronic tactile extinction. Front. Hum. Neurosci. 7:90. doi: 10.3389/fnhum.2013.00090, PMID: 23519604 PMC3602932

[ref95] Schmidt L. Keller I. Utz K. S. Artinger F. Stumpf O. Kerkhoff G. (2013b). Galvanic vestibular stimulation improves arm position sense in spatial neglect: a sham-stimulation-controlled study. Neurorehabil. Neural Repair 27, 497–506. doi: 10.1177/1545968312474117, PMID: 23401158

[ref96] Sprenger A. Spliethoff P. Rother M. Machner B. Helmchen C. (2020). Effects of perceptible and imperceptible galvanic vestibular stimulation on the postural control of patients with bilateral vestibulopathy. J. Neurol. 267, 2383–2397. doi: 10.1007/s00415-020-09852-x, PMID: 32350649

[ref97] Terney D. Chaieb L. Moliadze V. Antal A. Paulus W. (2008). Increasing human brain excitability by transcranial high-frequency random noise stimulation. J. Neurosci. 28, 14147–14155. doi: 10.1523/JNEUROSCI.4248-08.2008, PMID: 19109497 PMC6671476

[ref98] Thomas C. Truong D. Clark T. K. Datta A. (2020). Understanding current flow in galvanic vestibular stimulation: a computational study. Annu. Int. Conf. IEEE Eng. Med. Biol. Soc. 2020, 2442–2446. doi: 10.1109/EMBC44109.2020.9176716, PMID: 33018500

[ref99] Thompson T. L. Amedee R. (2009). Vertigo: a review of common peripheral and central vestibular disorders. Ochsner J. 9, 20–26, PMID: 21603405 PMC3096243

[ref100] Thompson S. L. O'Leary G. H. Austelle C. W. Gruber E. Kahn A. T. Manett A. J.. (2021). A review of parameter settings for invasive and non-invasive Vagus nerve stimulation (VNS) applied in neurological and psychiatric disorders. Front. Neurosci. 15:709436. doi: 10.3389/fnins.2021.709436, PMID: 34326720 PMC8313807

[ref101] Tohyama T. Kondo K. Otaka Y. (2021). Effects of galvanic vestibular stimulation on visual verticality and standing posture differ based on the polarity of the stimulation and hemispheric lesion side in patients with stroke. Front. Neurol. 12:768663. doi: 10.3389/fneur.2021.768663, PMID: 34858316 PMC8631773

[ref102] Tomioka Y. Tohyama T. Honaga K. Kawakami M. Kondo K. Tsuji T. (2022). Effects of galvanic vestibular stimulation on subjective visual vertical and sitting balance in patients with stroke. J. Stroke Cerebrovasc. Dis. 31:106430. doi: 10.1016/j.jstrokecerebrovasdis.2022.106430, PMID: 35279006

[ref103] Tran S. Shafiee M. Jones C. B. Garg S. Lee S. Pasman E. P.. (2018). Subthreshold stochastic vestibular stimulation induces complex multi-planar effects during standing in Parkinson’s disease. Brain Stimul. 11, 1180–1182. doi: 10.1016/j.brs.2018.04.020, PMID: 29776860

[ref104] Truong D. Q. Thomas C. Ira S. Valter Y. Clark T. Datta A. (2024). Unpacking galvanic vestibular stimulation using simulations and relating current flow to reported motions: comparison across common and specialized electrode placements. PLoS One 19:e0309007. doi: 10.1371/journal.pone.0309007, PMID: 39186497 PMC11346646

[ref105] Utz K. S. Keller I. Kardinal M. Kerkhoff G. (2011a). Galvanic vestibular stimulation reduces the pathological rightward line bisection error in neglect—a sham stimulation-controlled study. Neuropsychologia 49, 1219–1225. doi: 10.1016/j.neuropsychologia.2011.02.046, PMID: 21371483

[ref106] Utz K. S. Keller I. Artinger F. Stumpf O. Funk J. Kerkhoff G. (2011b). Multimodal and multispatial deficits of verticality perception in hemispatial neglect. Neuroscience 188, 68–79. doi: 10.1016/j.neuroscience.2011.04.068, PMID: 21596103

[ref107] Vallar G. (1997). Spatial frames of reference and somatosensory processing: a neuropsychological perspective. Philos. Trans. R. Soc. B Biol. Sci. 352, 1401–1409. doi: 10.1098/rstb.1997.0126, PMID: 9368928 PMC1692053

[ref108] Valter Y Moreno J Nazim K Gabay E Cohen S Clark T. (2021). “Galvanic vestibular stimulation headset balancing robust and simple administration with subject comfort: a usability analysis.” In *2021 43rd annual international conference of the IEEE engineering in Medicine & Biology Society (EMBC)*. pp. 5063-5066. IEEE. 10.1109/EMBC46164.2021.9630466 34892345

[ref700] Valter Y. Rapallo F. Burlando B. Crossen M. Baeken C. Datta A.. (2024). Efficacy of non-invasive brain stimulation and neuronavigation for major depressive disorder: a systematic review and meta-analysis. *Expert Rev. Med. Devices* 2, 643-658. doi: 10.1080/17434440.2024.2370820 38902968

[ref109] Volkening K. Kerkhoff G. Keller I. (2016). Effects of repetitive galvanic vestibular stimulation on spatial neglect and verticality perception—a randomised sham-controlled trial. Neuropsychol. Rehabil. 28, 1179–1196. doi: 10.1080/09602011.2016.1248446, PMID: 27820972

[ref110] Wardman D. L. Fitzpatrick R. C. (2002). What does galvanic vestibular stimulation stimulate? Adv. Exp. Med. Biol. 508, 119–128. doi: 10.1007/978-1-4615-0713-0_15, PMID: 12171101

[ref111] Weech S. Wall T. Barnett-Cowan M. (2020). Reduction of cybersickness during and immediately following noisy galvanic vestibular stimulation. Exp. Brain Res. 238, 427–437. doi: 10.1007/s00221-019-05718-5, PMID: 31938844

[ref112] Wiesenfeld K. Moss F. (1995). Stochastic resonance and the benefits of noise: from ice ages to crayfish and SQUIDs. Nature 373, 33–36. doi: 10.1038/373033a0, PMID: 7800036

[ref113] Wilkinson D. Ko P. Kilduff P. McGlinchey R. Milberg W. (2005). Improvement of a face perception deficit via subsensory galvanic vestibular stimulation. J. Int. Neuropsychol. Soc. 11:1076. doi: 10.1017/S1355617705051076, PMID: 16519272

[ref114] Wilkinson D. Zubko O. Sakel M. (2009). Safety of repeated sessions of galvanic vestibular stimulation following stroke: a single-case study. Brain Inj. 23, 841–845. doi: 10.1080/02699050903232541, PMID: 19697173

[ref115] Wilkinson D. Zubko O. DeGutis J. Milberg W. Potter J. (2010). Improvement of a figure copying deficit during subsensory galvanic vestibular stimulation. J. Neuropsychol. 4, 107–118. doi: 10.1348/174866409X468205, PMID: 19706224

[ref116] Wilkinson D. Zubko O. Sakel M. Coulton S. Higgins T. Pullicino P. (2014). Galvanic vestibular stimulation in hemi-spatial neglect. Front. Integr. Neurosci. 8:4. doi: 10.3389/fnint.2014.00004, PMID: 24523679 PMC3905204

[ref117] Woods A. J. Antal A. Bikson M. Boggio P. S. Brunoni A. R. Celnik P.. (2016). A technical guide to tDCS, and related non-invasive brain stimulation tools. Clin. Neurophysiol. 127, 1031–1048. doi: 10.1016/j.clinph.2015.11.012, PMID: 26652115 PMC4747791

[ref118] Wuehr M. Nusser E. Krafczyk S. Straube A. Brandt T. Jahn K.. (2016a). Noise-enhanced vestibular input improves dynamic walking stability in healthy subjects. Brain Stimul. 9, 109–116. doi: 10.1016/j.brs.2015.08.017, PMID: 26422129

[ref119] Wuehr M. Nusser E. Decker J. Krafczyk S. Straube A. Brandt T.. (2016b). Noisy vestibular stimulation improves dynamic walking stability in bilateral vestibulopathy. Neurology 86, 2196–2202. doi: 10.1212/WNL.0000000000002748, PMID: 27164706

[ref120] Wuehr M. Eder J. Keywan A. Jahn K. (2023). Noisy galvanic vestibular stimulation improves vestibular perception in bilateral vestibulopathy. J. Neurol. 270, 938–943. doi: 10.1007/s00415-022-11438-8, PMID: 36324034 PMC9886588

[ref121] Wuehr M. Eder J. Kellerer S. Amberger T. Jahn K. (2024a). Mechanisms underlying treatment effects of vestibular noise stimulation on postural instability in patients with bilateral vestibulopathy. J. Neurol. 271, 1408–1415. doi: 10.1007/s00415-023-12085-3, PMID: 37973635 PMC10896912

[ref122] Wuehr M. Peto D. Fietzek U. M. Katzdobler S. Nübling G. Zaganjori M.. (2024b). Low-intensity vestibular noise stimulation improves postural symptoms in progressive supranuclear palsy. J. Neurol. 271, 4577–4586. doi: 10.1007/s00415-024-12419-9, PMID: 38722328 PMC11233287

[ref123] Yamamoto Y. Struzik Z. R. Soma R. Ohashi K. Kwak S. (2005). Noisy vestibular stimulation improves autonomic and motor responsiveness in central neurodegenerative disorders. Ann. Neurol. 58, 175–181. doi: 10.1002/ana.20574, PMID: 16049932

[ref124] Zubko O. Wilkinson D. Langston D. Sakel M. (2013). The effect of repeated sessions of galvanic vestibular stimulation on target cancellation in visuo-spatial neglect: preliminary evidence from two cases. Brain Inj. 27, 613–619. doi: 10.3109/02699052.2013.767938, PMID: 23473288

